# Stretchable Sweat‐Activated Battery in Skin‐Integrated Electronics for Continuous Wireless Sweat Monitoring

**DOI:** 10.1002/advs.202104635

**Published:** 2022-01-28

**Authors:** Yiming Liu, Xingcan Huang, Jingkun Zhou, Chun Ki Yiu, Zhen Song, Wei Huang, Sina Khazaee Nejad, Hu Li, Tsz Hung Wong, Kuanming Yao, Ling Zhao, Woojung Yoo, Wooyoung Park, Jiyu Li, Ya Huang, Hiuwai Raymond Lam, Enming Song, Xu Guo, Yanwei Wang, Zhenxue Dai, Lingqian Chang, Wen Jung Li, Zhaoqian Xie, Xinge Yu

**Affiliations:** ^1^ Department of Biomedical Engineering City University of Hong Kong Kowloon Tong 999077 Hong Kong; ^2^ Hong Kong Center for Cerebra‐Cardiovascular Health Engineering Hong Kong Science Park New Territories 999077 Hong Kong; ^3^ State Key Laboratory of Structural Analysis for Industrial Equipment Department of Engineering Mechanics International Research Center for Computational Mechanics Dalian University of Technology Dalian 116024 China; ^4^ Ningbo Institute of Dalian University of Technology Dalian University of Technology Ningbo 315016 China; ^5^ Shanghai Frontiers Science Research Base of Intelligent Optoelectronics and Perception Institute of Optoelectronics Fudan University Shanghai 200433 China; ^6^ College of Construction Engineering Jilin University Changchun 130012 China; ^7^ Beijing Advanced Innovation Center for Biomedical Engineering School of Biological Science and Medical Engineering Beihang University Beijing 100083 China; ^8^ School of Biomedical Engineering Research and Engineering Center of Biomedical Materials Anhui Medical University Hefei 230032 China; ^9^ Department of Mechanical Engineering City University of Hong Kong Kowloon Tong Hong Kong

**Keywords:** microfluidic system, stretchable electronics, sweat‐activated batteries, sweat analysis, wireless communication

## Abstract

Wearable electronics have attracted extensive attentions over the past few years for their potential applications in health monitoring based on continuous data collection and real‐time wireless transmission, which highlights the importance of portable powering technologies. Batteries are the most used power source for wearable electronics, but unfortunately, they consist of hazardous materials and are bulky, which limit their incorporation into the state‐of‐art skin‐integrated electronics. Sweat‐activated biocompatible batteries offer a new powering strategy for skin‐like electronics. However, the capacity of the reported sweat‐activated batteries (SABs) cannot support real‐time data collection and wireless transmission. Focused on this issue, soft, biocompatible, SABs are developed that can be directly integrated on skin with a record high capacity of 42.5 mAh and power density of 7.46 mW cm^−2^ among the wearable sweat and body fluids activated batteries. The high performance SABs enable powering electronic devices for a long‐term duration, for instance, continuously lighting 120 lighting emitting diodes (LEDs) for over 5 h, and also offers the capability of powering Bluetooth wireless operation for real‐time recording of physiological signals for over 6 h. Demonstrations of the SABs for powering microfluidic system based sweat sensors are realized in this work, allowing real‐time monitoring of pH, glucose, and Na^+^ in sweat.

## Graphical abstract

A stretchable, conformable sweat‐activated battery (SAB) has been developed with high power density (7.46 mW cm^−2^) and energy capacity (42.5 mAh); it enables lighting 120 lighting emitting diodes (LEDs) for 5 h, and offers enough power to support Bluetooth wireless operation for real‐time recording of physiological signals in state‐of‐art wearable sensors for over 6 h. The SAB is also demonstrated in powering microfluidic system based sweat sensors for real‐time monitoring of pH, glucose, and Na^+^ in sweat.

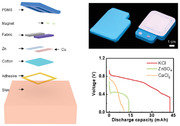

## 1 Introduction

Recently, thin and soft wearable electronics, sometime also known as skin electronics, have made significant progress in their electrical and mechanical properties and thus exhibit great potentials in healthcare monitoring, human machine interfaces, and clinical diagnosis.^[^ ^1^, ^2^, ^3^, ^4^, ^5^, ^6^, ^7^ ^]^ The power management unit is one of the most important components in these skin electronic devices, as it provides and regulates the electrical power for data collection, analysis, and transmission.^[^ ^8^, ^9^, ^10^, ^11^, ^12^, ^13^, ^14^, ^15^, ^16^, ^17^, ^18^ ^]^ Up to now, extensive powering technologies have been developed and used in wearable electronics, such as lithium‐ion (Li‐ion) batteries, self‐powered generators, bio‐fuel energy, and radio frequency (RF) based wireless powering strategies.^[^ ^19^, ^20^, ^21^, ^22^, ^23^, ^24^, ^25^, ^26^ ^]^ Unfortunately, these power sources are still incompetent for skin electronics due to their limitations in various aspects, such as the hazardous functional materials and rigid platform (Li‐ion battery),^[^ ^27^, ^28^ ^]^ low energy conversion efficiency (triboelectric, piezoelectric, and thermoelectric generators),^[^ ^21^, ^29^, ^30^ ^]^ unstable power outputs (biofuel cells),^[^ ^31^ ^]^ and ultrashort wireless transmission distance (RF technologies)^[^ ^32^ ^]^ (Table S1, Supporting Information). The lack of a general solution for powering skin electronics has pointed out the importance of developing advanced batteries in flexible or even stretchable formats. Wearable sweat‐activated batteries (SABs) offer a new powering strategy that are capable of generating abundant electricity from human sweat to power wearable devices for health monitoring.^[^ ^33^ ^]^ Moreover, adopting biocompatible functional materials allows SABs in flexible formats for long‐term interfacing with human skin or even inner organ tissues without secure risks.^[^ ^34^ ^]^ The reported flexible SABs have proven their capabilities to power some simple low power consumption electronic devices, however, they still suffer from low power outputs that significantly limit their applications in powering multifunctional skin electronics, especially the continuous wireless monitoring devices (Table S2, Supporting Information). Therefore, for the next‐generation power source in practical applications, SABs should meet the following requirements: (1) providing high power density and capacity for continuously and stably powering various portable and smart electronics; (2) exhibiting great mechanical flexibility and stretchability for the convenient integration with skin electronics and ensuring normal operation even under large deformations.

Here we report a class of materials, mechanical designs, and system integration strategies for soft, skin‐integrated, and stretchable SABs, that produce a record high capacity of 42.5 mAh and power density of 7.46 mW cm^−2^, and thus enables sufficient power for skin electronics to realize data collecting, processing, and wireless transmitting via gold standard Bluethooth technologies to continuously monitoring health related signals in sweat. Our SABs adopt biocompatible materials Zinc (Zn)^[^ ^35^ ^]^ and copper sulfate (CuSO_4_) embedded low‐cost water absorbable nylon fabric as the functional layer, and soft stretchable silicone based shell and soft cotton for isolating potential external disturbances as well as capturing sweat. Absorbing slight sweat as low as 0.06 mL cm^−2^ through the absorbent cotton in a single cell allows great and stable power output, which is sufficient to light over 120 lighting emitting diodes (LEDs) for over 5 h. Integration of four cells with soft microfluidic sweat sensing platform into a pack allows real‐time measuring of various sweat biomarkers of pH, Na^+^, and glucose, as well as continuous wireless data transmission via a Bluetooth module in the skin electronic device for over 6 h, which is comparable to the current Li‐ion batteries powered wearable electronics.

## 2 Results

**Figure 1:**
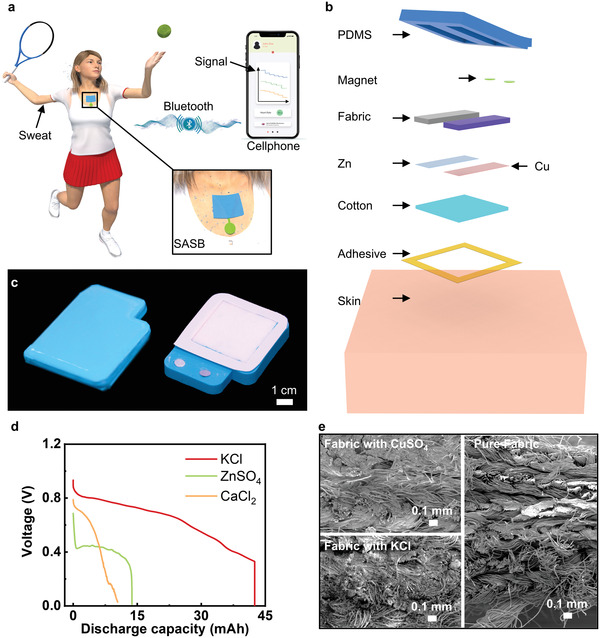
Materials and structures of the sweat‐activated battery (SAB). a) The 3D schematic illustration of a person wearing a flexible electronic device powered by the SAB for wireless sweat components monitoring. b). The exploded illustration of the SAB. c) Optical images of the top and bottom views of the SAB. d) Plots of discharge behaviors of the SAB with 20‐mL different saturated electrolytes, including KCl (red plot), ZnSO_4_(green plot), and CaCl_2_ (yellow plot). e). The scanning electron microscopy (SEM) images of the neat fabric and fabrics with CuSO_4_, KCl.

**Figure** **1**a shows a schematic illustration of a person wearing a skin‐integrated sweat sensing electronic powered by a SAB that allows data analyses and monitoring with long‐distance, real‐time wireless transmissions via Bluetooth to a smart phone. As shown in Figure 1b, Figures S1, and S2 (Supporting Information), the SAB consists of a soft silicone (polydimethylsiloxane, PDMS; moduli of 147 kPa) for fixing and loading functional materials, two magnets buttons (diameter, 5 mm; thickness, 1 mm) as two connection cables to the electrodes, two nylon fabric bags (each size, 3 × 1 × 2.5 mm) containing two kinds of biocompatible chemicals (KCl and CuSO_4_) as electrolyte, copper (Cu, 25 µm) and zinc (Zn, 80 µm) thin layers working as the cathode (Cu) and anode (Zn), a layer of hydrophilic cotton containing KCl powder (thickness, 1.6 mm; mass, 0.18 g cotton/0.22 g KCl) as salt bridge, and a strong waterproof adhesive layer at the bottom to enhance the bonding force with skin. Figure 1c shows the photos of a sweat‐activated flexible battery with top and bottom views, where the overall size of the SAB is 6.3 × 5 × 0.6 cm. The soft materials used in the SAB and the waterproof adhesive allow the SAB to be mounted onto different parts of the body for sweat collection up to 6 h without causing any skin irritation, where the highest Cu^2+^ concentration around the skin, 15 mg L^−1^, is much lower than the skin safety limit (10 g L^−1^) (Figures S3 and S4, Supporting Information).^[^ ^36^ ^]^ Figure S5 (Supporting Information) shows the working process of the SAB, where the sweat generated during exercise would be absorbed by the KCl embedded cotton, and immediately penetrating into the two fabric bags and thus dissolving the adherent chemicals. The absorption of sweat in KCl embedded cotton triggers the oxidation of Zn into Zn^2+^ (Equation 1) and the reduction of Cu^2+^ into Cu (Equation 2) at the same time. To maintain the electrical neutrality within the internal battery, Cl^–^ and K^+^ ions in cotton move to the KCl and CuSO_4_ fabric bags, respectively, for compensating the corresponding charge differences.

(1) $$\mathrm{Zn}\left(s\right)\to Zn^{2+}\left(\mathrm{aq}\right)+2e^{-}\left(\mathrm{Oxidation}\;\mathrm{half}\;\mathrm{reaction}\right)$$

In CuSO_4_ fabric bag, Cu^2+^ ions plate onto the copper metal sheet taking up electrons from the external circuit.

(2) $$Cu^{2+}\left(\mathrm{aq}\right)+2e^{-}\to \mathrm{Cu}\left(s\right)\;\left(\mathrm{Reduction}\;\mathrm{half}\;\mathrm{reaction}\right)$$

The overall reaction occurs between Zn and CuSO_4_.

(3) $$\mathrm{Zn}\;\left(s\right)+Cu^{2+}\left(\mathrm{aq}\right)\to Zn^{2+}\left(\mathrm{aq}\right)+\mathrm{Cu}\left(s\right)$$

As a result, the sweat absorbed by the cotton penetrates into the inner space of the battery that could realize the activation in a short time, and thus boost a constant voltage via the potential difference between oxidation and reduction reactions (Movie S1, Supporting Information). Table S3 (Supporting Information) summarizes the average estimated activated time of the SAB, where the experiment was conducted by users with different ages and weights to do various types of exercises. The activated duration can be as short as 28 min when users play American football. The activated time can be further decreased to 6 min as the SAB mounted onto the foreheads during moderate exercise intensity, as shown in Table S4 (Supporting Information).

**Figure 2:**
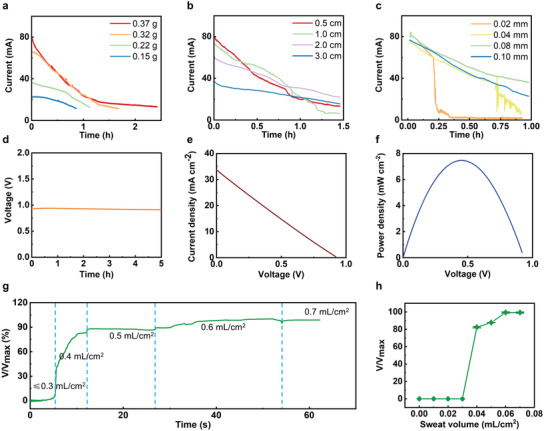
Electrical characteristics of the sweat‐activated battery (SAB). a) Output properties of the battery as a function of the CuSO_4_ contents. b) Output properties of the battery as a function of the space in between the two electrodes. c) Output properties of the battery as a function of the Zn foil thickness. d) Continuous output performance of the SAB in 5 h. e) Polarization characteristics of the SAB. f) Power density of the SAB as a function of the output voltages. g) The ratio of real time voltage output to maximum output voltage (*V*/*V*_max_) as a function of time with generally increasing the artificial sweat volume in the battery cell. h) *V*/*V*_max_ of the battery as a function of the added sweat volume.

3D printing creates the mold for the backbone of the SABs, pouring PDMS and demold forms the soft carrier, as shown in Figure S6 (Supporting Information). Two magnet sheets connected to the anode and cathode by metal wires are embedded in the soft carrier to obtain a detachable format (Figure S7, Supporting Information). Thin layers of metal sheets of Cu (thickness, 25 µm) and Zn (thickness, 80 µm) are attached to the fabric bags, which contain CuSO_4_ and KCl crystals, respectively. Subsequently, they have been fixed into the reserved positions in the carrier, followed by mounting a thick layer of cotton. PDMS sealed the sides of the cotton layer that prevents the leakage of the inner chemicals (Figure S8, Supporting Information). Figure S9 (Supporting Information) presents the electrical response of a representative SAB cell without any ionic chemical in cotton as a function of the injected artificial sweat (CAS, 1336‐21‐6).^[^ ^37^ ^]^ It is found that the highest open‐circuit voltage of the battery without ionic chemicals is only 0.55 V, far lower than the battery with KCl in cotton value (0.93 V) (**Figure** **2**d). Meanwhile, a higher concentration of the electrolyte solution (KCl) leads to a higher conductivity within the internal circuit of the battery, and thus offers greater power outputs. Figure 1d displays the capacities for cells with constant Zn and Cu sheets (thickness, 80 µm), CuSO_4_ immersed fabric bag (mass, 0.6 g), and three different fabric bags containing 0.37 g biocompatible, neutral KCl, calcium chloride (CaCl_2_), and zinc sulfate (ZnSO_4_), respectively. KCl fabric bag offers the best battery performance among all devices, with the highest capacity of 42.5 mAh as its voltage output drops from 0.93 to 0 V. To evaluate the Cu^2+^ diffusion rate, we recorded the Cu^2+^ concentration around Zn and Cu electrode. The results showed that the Cu^2+^ concentration is about 1 and 15 mg/L near Zn and Cu electrode after 6 h operation, where we can find loss of Cu is negligible that of the mass of the Cu electrode (Figure S3, Supporting Information). Figure 1e shows the scanning electron microscopy (SEM) images of the two chemically treated fabrics and an untreated neat one. By comparing to the neat nylon fabric, it is evident that two chemicals can crystalize and store in the fluffy space of the fabrics.

The electrical characteristics of the primary SABs are shown in Figure 2. The short‐circuit current of the SABs is shown in Figure 2a–c, and it is obvious that the output power density of the battery is directly related to CuSO_4_ contents (Figure 2a), the distance between two electrodes (Figure 2b), and the thickness of Zn foil (Figure 2c). The CuSO_4_ mass in the fabric bag would increase with the more cycles of immersing, reaching the maximum absorption value of 0.37 g after four cycles immersing. Accordingly, the duration would enhance from 1 to 2.5 h. Besides, it could be seen that the decreasing tendency of the four cycles immersed CuSO_4_ fabric bag is similar to that of the three cycles immersed fabric bag, indicating the CuSO_4_ mass in the fabric is close to saturation after three cycles immersion. In addition, the capacity of the SAB increases with the CuSO_4_ mass, ranging from 12.3 to 42.5 mAh as the mass of CuSO_4_ from 0.15 to 0.37 g (Figure S10a, Supporting Information). Hence, immersing the fabric bag four cycles is adequate for the wearable battery. Furthermore, the battery output is also affected by the distance between anode and cathode. The initial voltage of loads decreases with the increasing electrode distance, while the discharge rate has contrary tendency, which is related to the consumption rate of CuSO_4_ (Figure 2b). When the distance between the cathode and anode is 0.5, 1, and 2 cm, the SABs exhibit similar capacities around 43 mAh, while the capacity decreases to 34.8 mAh when the distance is 3 cm (Figure S10b, Supporting Information). For balancing outputs, working time, battery size and capacity, a distance between anode and cathode of 1 cm would be a good choice. Except for the CuSO_4_ content and the electrodes distance, the thickness of the Zn sheet is a significant impact factor for battery performance. As shown in Figure 2c, the operating time of the battery tends to be shorter as the Zn sheet is thinner. Likewise, the capacity of the SAB would increase with the increasing of the thickness of the Zn sheet, ranging from 11.8 to 42.5 mAh (Figure S10c, Supporting Information). This phenomenon is due to the rapid consumption of Zn, as which acts as the reactant in the battery (Equation 1). However, there is no noticeable difference in the output performance of the battery with a Zn different sheet thickness of 100 and 80 µm, indicating the Zn sheet thickness of 80 µm is suitable for this battery. Furthermore, the effects of pH and ambient temperature are investigated, and the results are shown in Figure S10d,e (Supporting Information). The experiments were carried out by using the artificial sweat with different pH as the electrolyte at room temperature to study the capacity variations, where we found the capacity of SABs increase with the decreases of pH values because of the presence of hydrogen ions^[^ ^34^ ^]^ (Figure S9d, Supporting Information). In addition, loss of capacity in SABs is observed as the increasing of ambient temperature (Figure S10e, Supporting Information). This phenomenon is caused by increases of evaporation rates under low‐flow‐rate conditions, resulting in increased internal resistance (*R*s).^[^ ^34^ ^]^ As a result, under the optimal configuration, the SAB can continuously deliver an open circuit voltage (OCV) over 0.93 V for over 6 h (Figure 2d). The polarization in Figure 2e shows that the maximum current density (MPC) could reach 33.75 mA cm^–2^. Accordingly, the maximum power density (MPD) is calculated as 7.46 mW cm^–2^, high output for SABs.

**Figure 3:**
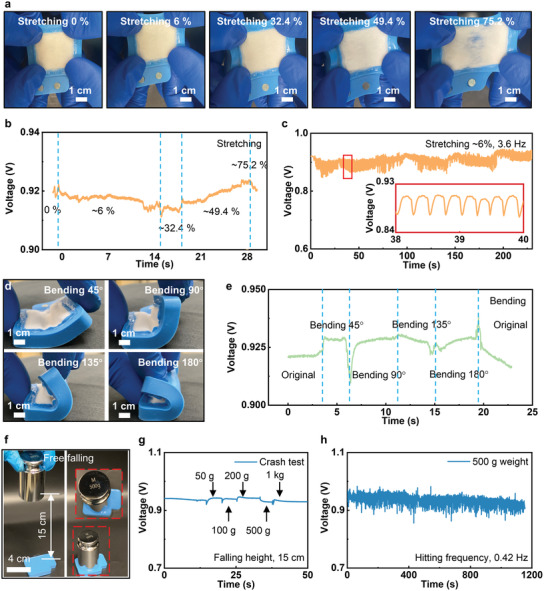
Mechanical characteristics of the sweat‐activated battery (SAB). a,b) Optical images of the SAB as stretched to various rates, including 0, 6%, 32.4%, 49.4%, and 75.2%, and the corresponding voltage outputs, fluctuating between 0.91 and 0.93 V. c) Electrical response of the SAB during repeated cycles (>800) of stretching ≈6% at a constant frequency of 3.6 Hz. d,e) Optical images of the battery bent at different angles from 45° to 180°, and the corresponding electrical responses. f) Optical images of the crash test setup. g) Electrical response of the battery as the five different weights hit it from a constant height of 15 cm, as shown in (f). h) Electrical response of the battery as the 500 g weight continuously hits it 500 times at a constant frequency of ≈0.42 Hz.

To investigate the minimum sweat volume (MSV) for activating the SAB, the controlled volume of artificial sweat is added into the battery, as shown in Figure 2g. It is obvious that the MSV value of 0.04 mL cm^−2^ yields ≈82% of the highest OCV of the battery, 0.05 mL cm^−2^ @87% OCV, and 0.06 mL cm^−2^ @99% OCV (Figure 2h). So, we can conclude 0.06 mL cm^−2^ is the MSV that can fully activate the SAB. **Figure** **3**a and Figure S11 (Supporting Information) show the optical images of a SAB under various stretching rates, ranging from 0% to 75.2%, and it is obvious that the battery starts disassembling at the maximum stretching rate. Figure 3b presents the electrical response of the battery under the four different stretching rates, ranging from 6% to 75.2%. In the beginning, the OCV by the battery fluctuates from 0.91 to 0.93 V, exhibiting its stable electrical characteristics. Until ≈75.2% stretching rate, the battery breaks down, resulting in the OCV sharp drop. In addition, the OCV of the SAB stabilizes between 0.85 and 0.93 V after 800 cycles stretching at a constant rate and frequency of ≈6% and 3.6 Hz (Figure 3c). Figure 3d presents the photos of the SAB under four different bending angles, including 45°, 90°, 135°, and 180° folding. The OCV of the battery exhibits a stable value (0.92–0.94 V) as bent from 0° to 180° (Figure 3e). Additionally, the SAB presents stable electrical characteristics with a small fluctuation of 0.05 V as bent at 135° under a frequency of 4 Hz (Figure S12, Supporting Information). Figures 3f,g presents a crash test of the SAB, where various weights from 50 g to 1 kg hitting the SAB through free falling wouldn't cause output voltage degradation, indicating the robust performance and excellent safety. Furthermore, repeating crashing test associates with 500 g weight continuously hits the SAB for over 500 times, showing negligible voltage output decay, further proving the excellent mechanical stability of the battery in practical applications (Figure 3h). Figure S13 (Supporting Information) shows the voltage output of the SAB as a function of the connected resistance load, ranging from 2.5 Ω to 10 kΩ, corresponding to 0.20 to 0.93 V, which indicates the loading of battery would be also an important factor for practical use.

**Figure 4:**
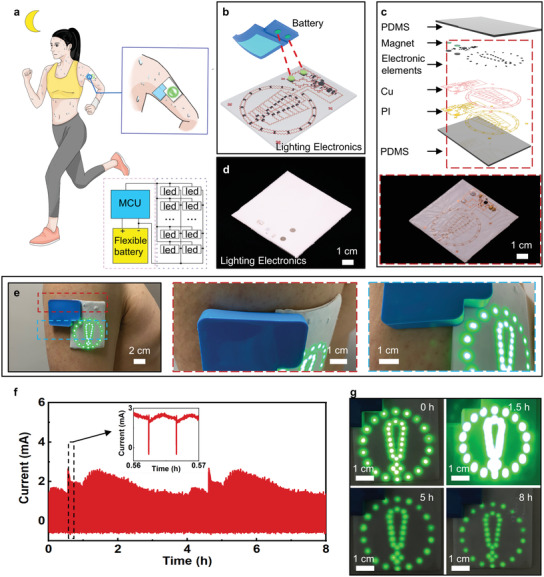
Demonstrations of the sweat‐activated battery (SAB) in powering flexible lighting electronics for safety night running. a) The schematic diagram of a runner wearing a sweat‐activated flexible battery powered LED warning sign for safety night running. b) The schematic diagram of the flexible lighting electronics powered by the removable battery. c) The exploded view of the flexible lighting electronics. d) Optical image of the lighting electronics with a thin polydimethylsiloxane (PDMS) layer encapsulation on top. e) Optical images of the lighting electronics with the sweat‐activated battery cell mounted onto a volunteer's upper arm. f) Current flow to the LED array with under a stabilized voltage of 3 V. g) Optical images of the lighting electronics powered by the flexible battery at 0, 1.5, 5, and 8 h.

An exemplary application of the SAB is to power a stretchable lighting electronics. Here, a human subject is wearing the stretchable lighting electronics powered by the SAB for evening running (**Figure** **4**a and Movie S2, Supporting Information). The generated sweat around the upper arm can activate the battery. Then the lighting electronics are turned on to alarm others for the runner's safety. Figure 4b shows the schematic diagram of the detachable connection method between the SAB and the lighting electronics through the embedded magnets. The lighting electronics consists of two encapsulation layers (PDMS, 1:30), two magnets (diameter, 5 mm; thickness, 1 mm), and a collection of chip‐scale integrated circuits (resistors, capacitors, energy harvesting module, and chip‐size LEDs) connected by filamentary serpentine Cu metallic traces (Figure 4c). Figure 4d shows the photo of a stretchable lighting electronics after encapsulation. Figure 4e shows the photo of a lighting electronic device and the SAB connected together mounting on an experimenter's upper arm. The enlarged details in Figure 4e verify the great contact behavior between the whole soft system and the human skin surface. The finite element analysis (FEA) results indicate that equivalent strain in the Cu layers of the lighting electronics is less than the yield strain (0.3%) for up to 90° twisting and 180° bending at radius of curvature of 2.5 mm, and significantly less the fracture strain (≈5%) for 20% stretching (Figure S14, Supporting Information). These results highlight the range of robust to accommodate realistic physiological motions on the underlying skin. To further demonstrate the electrical performance of the SAB, we measured the current output of the SAB as powering the lighting electronics after injecting 1 mL artificial sweat into the battery, as shown in Figure 4f. The current output reaches the highest value (2.5 mA) at 1.5 h, leading to the highest power density (7.5 mW), as verified in Figure 4g. Then, the power output of the SAB tends to drop from 1.5 to 8 h, resulting in the obvious decreasing of the lighting intensity by the flexible integrated‐lighting device. The long operating duration of the SAB (≈8 h) ensures the sufficient lifetime for users. Besides the flexible lighting electronics, the SAB can continuously lighten a 120‐LED electronics for over 5 h (Figure S15 and Movie S1, Supporting Information), further demonstrating the high power output and stored energy.

**Figure 5:**
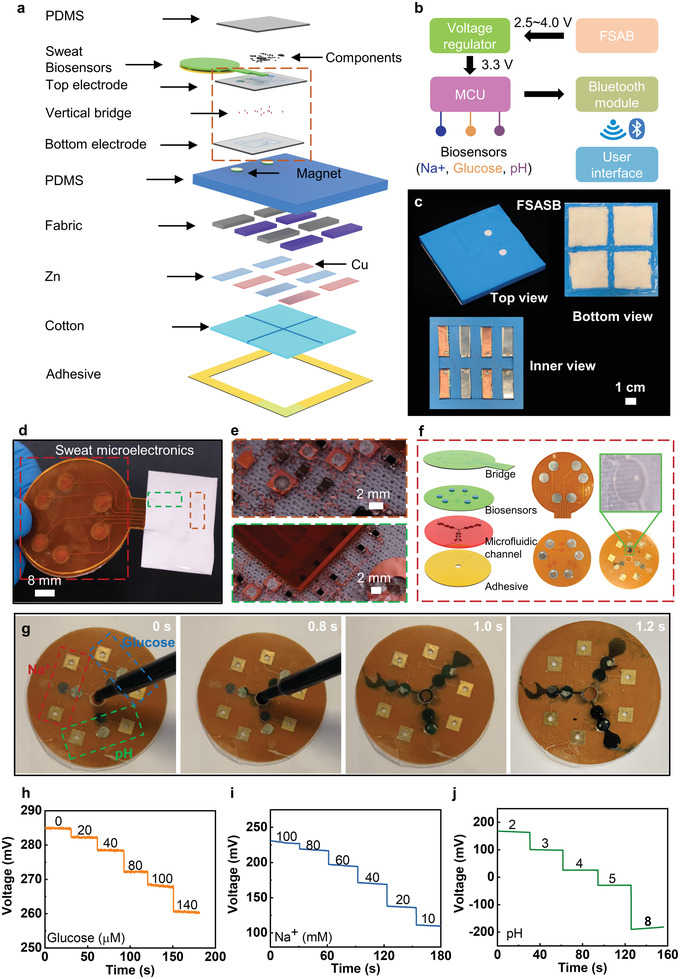
Wireless, skin‐integrated, microelectronic system for continuous sweat monitoring, powered by a sweat‐activated battery array. a) Schematic diagram of the exploded view of the wireless, flexible microelectronic system with the sweat‐activated battery cells for long‐term monitoring Na^+^, glucose, and pH value of sweat. b) Schematic diagram of the whole system with the microelectronic system and four integrated battery cells. c) Optical image of the four integrated sweat‐activated battery cells connected in series with the top, bottom, and inner views. d) Optical images of the microelectronic systems with detachable microfluidic system. e) Optical images of the enlarged details of the circuit. f) Exploded‐view schematic diagram of the replaceable biosensors with optical images of each layer. g) Process of the microfluidic system absorbing colored water drop. h‐j) Open circuit voltage (OCV) responses of the glucose, Na^+^ and pH sensors.

To prove the high power density output and the capacitance of the SAB, we demonstrate a representative application that associates with using four stretchable battery cells connected in series to power sweat microelectronics directly. The smart sweat microelectronics is capable of collecting biomarkers related data in sweat through a self‐developed microfluidic system. Then, it wirelessly transmits data to a paired cellphone via Bluetooth in real‐time. **Figure** **5**a and Figure S16 (Supporting Information) show the schematic diagrams of the four packed SABs‐powered sweat microelectronics (FSAB‐SE). The whole system consists of three parts: 1) a stretchable integrated circuit, incorporating a Bluetooth module for data collection, analysis, and long‐range wireless transmission; 2) flexible sweat biosensors for pH, Na^+^, and glucose concentrations measurement and a microfluidic system for spontaneously collecting sweat into the target biosensors with controlled flow rate and single directions; 3) four SAB cells connected in series for boosting a high OCV and sufficient power density for the whole system. Figure 5b and Figure S17 (Supporting Information) show the circuit design of the whole system. Before the SABs activated by sweat, a pre‐charged supercapacitor can be added in the system on to power the microelectronics for up to couple of minutes (Figure S18, Supporting Information). Once activated by sweat, the SABs yield a high power density (3.2 V, ≈64 mW). Then the voltage regulator helps to stabilize the voltage output around 3.3 V, sufficient to normally operate the microcontroller (MCU) and other electronics. The data collected from three types of biosensors (Na^+^, glucose, and pH), is further converted into a 2‐byte value by the MCU and then send for Bluetooth transmission. Here, the outputs from Na^+^ sensor and pH sensor would pass through a voltage divider circuit and be recorded by an analog to digital converter (ADC) on the microcontroller while the positive and negative output of the glucose sensor would be amplified 10 times by an amplifier before recorded by the ADC. The receiving system (paired cellphone) translates the 2‐byte data, then displays it in real‐time mode. Figure 5c shows the optical images of the SABs with its top, bottom, and inner views, where two magnets on the top surface of the packed battery ensure strong physical contact with the above soft sweat microelectronics. Figure 5d and Figure S19 (Supporting Information) show the photos of the sweat microelectronic device, where the stretchable circuit adopts two layers of filamentary serpentine Cu metallic circuits based on well‐established mechanics design rules,^[^ ^38^, ^39^, ^40^ ^]^ and vertically connected through silver paste at pre‐formed holes (Figure 5e and Figures S20–S22). To passively gather sweat into the target biosensors, a self‐developed microfluidic system is attached onto a flexible platform where three biosensors have been integrated, as shown in Figure 5f. Six magnets at the opposite side of the biosensors are electrically connected with the bridge extended from the integrated circuit for data collection in a detachable form (Figure S19, Supporting Information). Figure 5g and Movie S3 (Supporting Information) demonstrate a drop of colored artificial sweat falling into the inlet of the microfluidic system for spontaneously pulling the sweat to the target biosensors in a short time. FEA results show that equivalent strain in the Cu layers of the sweat microelectronics is less than the yield strain (0.3%) for up to 50° twisting and 180° bending at a radius of curvature of 2.5 mm, and significantly less the fracture strain (≈5%) for 20% stretching (Figure S22, Supporting Information). These results show the high levels of the flexibility and stretchability of the device owing to its overall layouts, with an ability to accommodate extreme deformations of the underlying skin. Figure S23 (Supporting Information) presents the wireless communication distance between the sweat microelectronics and the coupled cellphone and demonstrates that the SABs are able to keep powering the electronics for over 6.3 h. It is found that the Bluetooth transmission distance stabilized at 6.38 m, sufficient for sweat microelectronics communicating with the coupled cellphone.

Figure 5h–j shows the performance of the flexible biosensors for sweat monitoring. In order to simplify the design of the circuit to detect and transmit sensing signals, all sensors are designed as potentiometric sensors without any transimpedance amplifiers. Here, the layer‐by‐layer structure is constructed for flexible biosensors. For the glucose sensor, glucose oxidase (GOx) could oxidize glucose as glucolactone and hydrogen peroxide (H_2_O_2_) firstly. And then the platinum nanoparticles on the electrode would reduce the produced H_2_O_2_ to generate electricity signals. The monitoring for Na^+^ and pH levels is achieved by the ion‐selective electrodes (ISEs). As a result, the potentiometric sensors can be obtained for long‐term continuous measurements coupled with reference electrodes.^[^ ^3^, ^20^, ^41^ ^]^ In Figure 5h and Figure S24a (Supporting Information), it could be seen that the voltage of the glucose sensors (versus Ag/AgCl) would decrease with the increase of glucose concentration. Besides, the voltages of the glucose sensors are linearly proportional to the glucose concentrations with a determination coefficient (*R* ^2^) of 0.998. At the same time, the anti‐interference ability and selectivity are shown in Figure S25a (Supporting Information). The voltage changes are apparent when glucose solution is added into the electrolyte, and the response is linearly related to the glucose concentration. No obvious changes of sensing signals can be observed when 20 × 10^−6^ msodium chloride (NaCl) and 20 × 10^−6^ mpotassium chloride (KCl) were added, indicating great selectivity and anti‐interference ability of the sensor. The response behaviors of the Na^+^ sensor versus physiologically relevant concentration range from 10 to 100 × 10^−3^ mare shown in Figure 5i and Figure S24b (Supporting Information). An excellent linear relationship between the OCV (versus Ag/AgCl) of the Na^+^ sensor and the value of the logarithm of Na^+^ concentration, with an excellent *R* ^2^ of 0.994 that corresponds to a sensitivity of 131.95 mV per decade of concentration for Na^+^. Figure S25b (Supporting Information) shows the study of the selectivity of the Na^+^ sensor, where the sensor exhibits negligible voltage fluctuations when glucose and KCl were added, indicating great selectivity. Figure 5j and Figure S24c (Supporting Information) show the voltage responses of the pH sensor to various pH value from 2 to 8, and the corresponding calibration plots. A near‐Nernstian sensitivity of 58.27 mV per pH is observed for the pH sensor based on polyaniline (PANI), with an *R* ^2^ of 0.995. This sensor also shows good selectivity and anti‐interference ability (Figure S25c, Supporting Information), as the sensor is only responsive to H^+^ concentration. In conclusion, all these soft biosensors exhibit good performance, including linearity, sensitivity, and selectivity, which would be extremely important for real‐time and continuous analysis of perspiration contents and reflecting the health status of the testing subjects.

**Figure 6:**
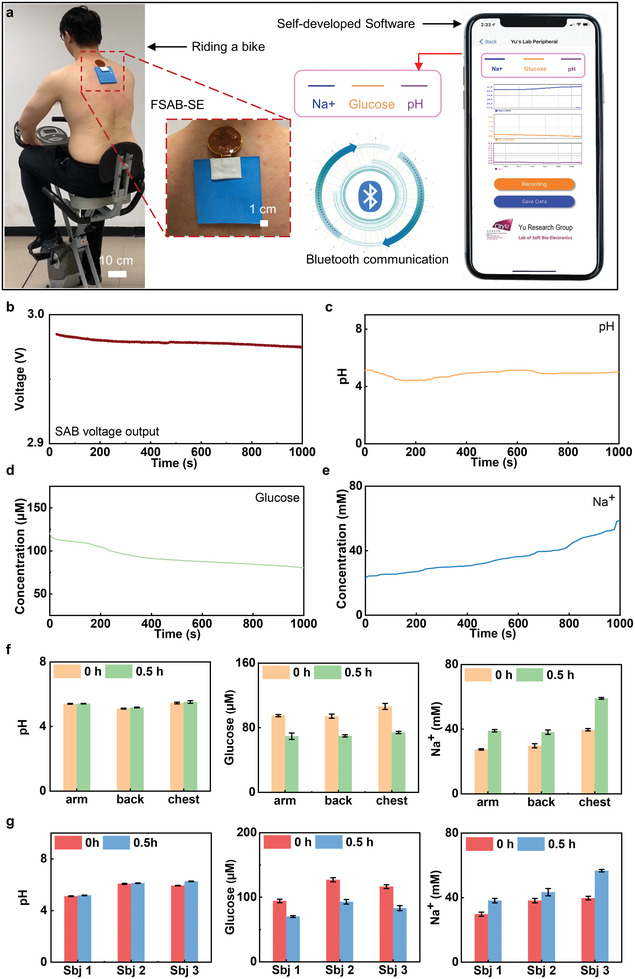
On‐body real‐time perspiration analysis and Bluetooth data transmission powered by the sweat‐activated battery. a) Optical images of a subject wearing the battery powered sweat sensing system to exercise while wirelessly monitoring Na^+^, glucose concentrations, and pH levels via Bluetooth and in real‐time displayed on a smart phone. b–e) The voltage output of the sweat‐activated batteries (SABs) as powering the microelectronics and the real‐time sweat analysis results of the concentration of pH, glucose, and Na^+^ biosensors during exercising. f) The comparison of concentration changes during exercise for three different body locations, including arm, back, and chest. g) The comparison of concentration changes during exercise on backs of three different subjects.

**Figures** **6**a and Movie S4 (Supporting Information) show a volunteer wearing SABs integrated sweat sensors on the back for continuous metabolic monitoring during a drastic cycling exercise. The measured biomarker information is wirelessly transmitted to the coupled cellphone for real‐time display. Figure 6b–e shows the voltage output and the data collected from the SABs integrated sweat sensors for real‐time monitoring of Na^+^, pH, glucose during a representative 1000 s. While the subject is cycling, the Na^+^ level in sweat increases rapidly due to the increasing volume of generated sweat of the experimenter. At the beginning of 300 s, glucose concentration decreased from 122 to 100 × 10^−3^ m and then stabilized over time, as the increasing sweat rate could dilute their concentrations. While the pH maintain a stable value of 4.9 through the exercise, which is consistent with the results in previous studies.^[^ ^3^, ^42^, ^43^ ^]^ Figure 6f and Figure S26 (Supporting Information) demonstrate sweat Na^+^, glucose, and pH levels measured 0.5 h after continuous cycling exercise, compared with the initial levels, in the three different body areas, including arm, back, and chest. It is evident that the Na^+^ arises, glucose declines, and pH stabilizes after 0.5 h drastic exercise despite of body locations. Then, we measured the Na^+^, glucose, and pH levels variations of three subjects on their backs, and similar trends were observed for these biochemical signals in different subjects, indicating the universality of the sensing sensor (Figure 6g). The results highlight the potential of SABs‐powered microelectronic systems for simultaneously capturing monitoring biochemical information for comprehensive biochemical signals analysis in sweat. Benefited from the long operating duration and stable power output, the SABs have presented a great potential as practical, biocompatible power sources for skin‐integrated electronics.

## 3 Conclusion 

In this study, we developed soft, stretchable SABs based on the chemical reaction between Zn and CuSO_4_. The reported batteries exhibit a record high power density and energy capacitance. A slight sweat volume (0.04 mL cm^−2^) can trigger the activation of the batteries rapidly. Careful selection of biocompatible functional materials in the batteries ensures long‐term lamination onto the human skin surface without any chemical hazard. Advanced mechanics designs allow the batteries exhibiting stable electrical outputs under various types of deformations, for instance maintaining unchanged electrical performance under 49.4% stretching. Demonstrations of the SAB associating with powering over hundreds of LEDs for 5 h by sweat for safety night running prove the high energy capacitance for practical power managements. Furthermore, combining four SAB cells in series enable direct powering of a self‐developed integrated circuit for continuous biochemical signals monitoring in sweat, including Na^+^, glucose, and pH levels via Bluetooth wireless transmission of sensor data. The results are very promising for advancing stretchable electronics for a wide range of applications, such as healthcare monitoring, human machine interfaces, and human life support.

## 4 Experimental Section

### Fabrication of the SAB

The fabrication process is represented in Figure S6 (Supporting Information). First, a rigid mold was printed using a 3D printer. A 3D software, Solidworks, was used to design the model, and resin was used as the printing material. Second, the printed mold had been used as a container to cure PDMS in the desired shape. After PDMS hardens, the flexible case for the battery was ready. Two small magnets were embedded in the reserved position while connected to the battery's electrodes in the next step. The process is represented in Figure S7 (Supporting Information). In parallel, two fabric bags had been immersed in the saturated CuSO_4_ and KCl solutions, and dried at 120 °C for 10 min. Repeat the former step until the fabric bags achieving designed masses. Then, they were attached to two Zn and Cu metal sheets. According to the battery working principle, the Cu sheet, integrated with CuSO_4_ fabric, acted as the cathode. Similarly, the Zn sheet, integrated with KCl fabric, worked as the anode. Subsequently, these two metal sheets and fabric bags were fixed in the reserved places in the flexible carrier. Eventually, water‐absorbable cotton with KCl powder, to absorb sweat, was placed on top of the metal sheets. Furthermore, silicon sealed four sides of the cotton layer in order to prevent inner ions leakage. The weights of the SAB and FSAB were 11.8 and 38.65 g, respectively.

### Assembly of the Lighting Electronics

The fabrication started on a quartz glass, which was first cleaned by acetone, alcohol, and deionized water (DI water) sequentially. Sodium stearate aqueous solution was spin coated on the glass sheet, serving as a thin sacrificial layer, then dried under 100 °C for 5 min for later releasing the device. Then, spin‐coating a thin PDMS film (0.17 mm) at 600 rpm for 30 s, baked at 110 °C for 5 min formed the stretchable substrate for the lighting electronics. To ensure enough adhesion strength between the Cu circuit layer and the PDMS substrate, another ultrathin PDMS film was spread over the cured PDMS substrate before attaching PI supported Cu film on it. After smoothly attaching the PI (12 µm) supported Cu (6 µm) sheet on the PDMS substrate, curing the sample at 110 °C for 5 min, and then patterned by photolithography and etching yielding metal traces in the desired geometries. Here a positive photoresist (PR, AZ 4620, AZ Electronic Materials) was spin‐coated at 3000 rpm for 30 s, soft bake on a hot plate at 110 °C for 5 min, then exposed to ultraviolet light for 45 s, and finally developed for 1 min in a solution (AZ 400K). After development, the PR was removed by acetone and rinsed by DI water. Low temperature solder joints bonded and electrically connected all of the components, including the energy harvesting module (TI, BQ25504), bridge wires (resistance, 0 Ω), capacitors (0.1≈100 µF), resistors (1.43≈10 MΩ), inductance (22 µH), and the patch type LEDs (1 × 0.5 mm) to corresponding contact pads on the Cu/PI substrate. Finally, pour PDMS (145 kPa, 0.17 mm thick) onto the electronics followed by curing at 110 °C for 5 min encapsulated the device. The skin‐integrated lighting electronics can be worn on the skin for extended periods, with various levels of physical activity, without irritation (Figure 4e). The weight of the lighting electronics was 6.6 g.

### Assembly of the Integrated Circuit of the Sweat Microelectronics

The first circuit layer fabrication process of the sweat microelectronics is same as that of the lighting electronics. After obtaining the first circuit layer, a thin layer of PDMS (50 µm, 1000 rpm for 60 s) was spin coated onto the patterned Cu traces, then attached another PI supported Cu film on it with markers of the first circuit layer exposing for later alignment. Curing the sample at 110 °C for 5 min, and then patterned by photolithography and etching yielding metal traces in the desired geometries. Here a positive photoresist (PR, AZ 4620, AZ Electronic Materials) was spin‐coated at 3000 rpm for 30 s, soft bake on a hot plate at 110 °C for 5 min, then exposed to ultraviolet light for 45 s, and finally developed for 1 min in a solution (AZ 400K). After development, the PR was removed by acetone and rinsed by DI water. Mechanically tear off the thin PDMS at the vertical bridge locations between the top and bottom metal traces through a high‐precision tweezer, then fill in silver paste for realizing the vertical bridge (Figures S19 and S20, Supporting Information). Low temperature solder joints bonded and electrically connected all of the components, including the microcontroller (TI, CC2640R2F), antenna (2450AT18B100), voltage regulator (TI, TPS76933), amplifier (TI, INA321), capacitors (0.1≈10 µF), resistors (1 kΩ ≈2 MΩ), inductance (10 µH), and a bridge (electrically connecting the biosensors to the integrated circuit) to corresponding contact pads on the Cu/PI substrate. Finally, pour PDMS (145 kPa, 0.17 mm thick) onto the electronics followed by curing at 110 °C for 5 min encapsulated the device. The weight of the integrated circuit is 1.33 g.

### Fabrication of the Biosensors of the Sweat Microelectronics

SU8 2050 photoresist (MicroChem, USA) was first spin‐coated onto the silicon wafer at 1150 rpm for 30 s, after obtained SU‐8 master with a depth of 160 µm, UV exposure, and development were processed as the conventional lithography fabrication process; PDMS precursor (elastomer base: curing agent = 10:1, SylgardTM 184, Dow Corning, USA) was poured onto the SU8 master mould, cured at 80 °C for 12 h, and peeled off. The peeled off PDMS was as for the second mask, Norland optical adhesive 63 (NOA63, Norland Products, USA) was adopted as for the microchannel material in our wearable device since its hydrophilic property compared to PDMS. Uncured NOA63 glue was poured into the surface of second PDMS master then with a commercially available polypropylene film for UV‐curing for 60 s, then the microchannel layer was formed for punching, commercially solid gum was used for joining this layer to the electrodes PI layer.

Fabrication of the flexible biosensors array began with a PI film (thickness 25 µm) bonded to copper foil (thickness 18 µm) on each side. Pattern and circuit were fabricated by printed circuit board processing techniques of copper (thickness 10 µm) plated with gold (thickness 50 nm). Furthermore, the cover layer was applied to both sides with exposing circle electrodes and square patches. For the fabrication of reference electrodes, Ag/AgCl paste was screen printed on the gold electrodes with a thickness of 100 µm. For the fabrication of potentiometric glucose sensors, platinum black was firstly electrodeposited on the gold electrode with a constant voltage of −0.8 V for 180 s. The electrolyte was composed by 24 × 10^−3^ m L^−1^ chloroplatinic acid and 2.1 × 10^−3^ m L^−1^ lead acetate. And then the mixture of 2 µL glucose oxidase (2.5 UµL^−1^), 2 µL chitosan (1% weight by weight, w/w) and 2 µL glutaraldehyde (2% w/w) was covered on platinum black membrane as the catalyst. To prepare Na^+^ selective electrode, poly(3,4‐ethylenedioxythiophene) polystyrene sulfonate (PEDOT: PSS) film was firstly electropolymerized on the gold electrode with a constant current of 0.2515 mA for 2856 s.^[^ ^44^ ^]^ The mixed solution of 0.01 m L^−1^ 3,4‐ethylenedioxythiophene (EDOT) and 0.1 m L^−1^ polystyrene sulfonate (NaPSS) was applied as the electrolyte. Then, 10 µL Na^+^ selective membrane cocktail was dropped on the PEDOT film. Na^+^ selective membrane cocktail consisted of Na ionophore X (1% w/w), sodium tetrakis [3,5‐bis(trifluoromethyl)phenyl] borate (Na‐TFPB, 0.55% w/w), polyvinyl chloride (PVC, K‐value 72‐1, 33% w/w), and bis(2‐ethylehexyl) sebacate (DOS, 65.45% w/w).^[^ ^3^, ^45^, ^46^ ^]^ And 200 mg of the cocktail was dissolved in 1320 µL of tetrahydrofuran17. Besides, the PVB reference electrode was modified by dropping 10 µL polyvinyl butyral (PVB) reference electrode solution on the Ag/AgCl electrode, which was prepared by dissolving 79.1 mg PVB, 50 mg of NaCl, 2 mg bag polymer PEO‐PPO‐PEO (F127), and 0.2 mg of multiwall carbon nanotubes into 1 mL methanol. For the fabrication of pH sensitive electrode, PANI was electrodeposited on the gold electrode by cyclic voltammetry method from −0.1 to 0.95 V with a scan speed of 50 mV s^−1^. The mixture of 0.1 m L^−1^ aniline, 0.5 m L^−1^ hydrochloric acid and 1 m L^−1^ sulfuric acid was selected as the electrolyte. Electrodeposition and electropolymerization were both achieved by electrochemical station of CHI 760E in a three‐electrode system. Thereinto, the gold electrode, Ag/AgCl electrode and platinum wire are the working electrode, reference electrode, and counter electrode, respectively. The weight of the biosensors system was 5.3 g.

### Characterization

Figure 1d was measured by electrochemical station of CHI 760E with a constant discharge current of 1 mA cm^−2^. In Figure 1e, SEM images were obtained by FEI Quanta 250. Figure 2a–c is measured by connecting a 2.5 Ω resistance to the battery, then measure the voltages of the 2.5 Ω resistance by PL3516/P 16/35 with a constant sampling frequency of 400 Hz. Figure 2d–f was measured by Keysight B1500A semiconductor analyzer. The polarization curve was obtained by *I*/*V* analysis from OCV to zero, the current density was calculated through the reaction area of electrodes (3 cm^2^) and the power density was the product of voltage and current density. Figures 2a–c,g,h, 4f, and 5h–j were measured by a PL3516/P 16/35 with a constant sampling frequency of 400 Hz.

### Mechanical Simulation

The FEA commercial software ABAQUS was used to optimize the shapes of interconnects and the layouts of functional components for improving the mechanical characteristics of the devices. The objective was to decrease the strain level in copper interconnects under different typical loads, including stretching, bending, and twisting. The PDMS was modeled by hexahedron elements (C3D8R) while the thin copper (18 µm thick) and PI (25 µm thick) layers were modeled by shell elements (S4R). The minimal element size was 1/4^th^ of the minimum width of interconnects (150 µm), which ensured the convergence and the accuracy of the simulation results. The elastic modulus (*E*) and Poisson's ratio (*ν*) used in the analysis were *E*_Cu_ = 119 GPa and *ν*_Cu_ = 0.34 for copper; *E*_PI_ = 2.1 MPa and *ν*_PI_ = 0.34 for PI; *E*_PDMS_ = 145 kPa and *ν*_PDMS_ = 0.5 for PDMS; *E*_magnet_ = 160 GPa and *ν*_magnet_ = 0.25 for magnet.

### Statistical Analysis

The illustration models were constructed on a Rhino software (Robert McNeel & Associates). The SEM images were obtained by FEI Quanta 250. Data was mainly obtained by the data acquisition (DAQ)/multimeter system (PowerLab 16/35, AD Instruments) and semiconductor analyzer (Keysight B1500A). Data processing was operated by the Origin software (OriginLab). FEA was operated by commercial software ABAQUS.

## Conflict of Interest

The authors declare no conflict of interest.

## Author Contributions

Y.L., X.H., J.Z., C.Y., and Z.S. contributed equally to this work. Y.L., X.H., Z.D., W.L., L.C., and X.Y. conceived the ideas, and designed the experiments. Y.L., Z.X., X.Y., X.H., C.Y., W.H., E.S., and S.N. wrote the manuscript. Y.L., X.H., C.Y., J.Z., W.H., H.L., T.W., K.Y., W.Y., W.Y., W.P., L.Z., J.L., JY.L., and HR.L. performed experiments. Y.L., X.H., Y.H., and Y.W. analyzed the experimental data. Z.X., X.G., and Z.S. performed structural designs, mechanical and electromagnetic modelling. J.Z., C.Y., and S.N. performed circuit designs.

## Code Availability

Custom code used in this study is availability from the corresponding authors upon reasonable request.

## Supporting information

Supporting Information

Supplemental Movie 1

Supplemental Movie 2

Supplemental Movie 3

Supplemental Movie 4

## Data Availability

The data that support the findings of this study are available from the corresponding author upon reasonable request.
