# Supplementary material for: Stretchable Sweat‐Activated Battery in Skin‐Integrated Electronics for Continuous Wireless Sweat Monitoring
Source: Adv Sci (Weinh). 2022 Jan 28;9(9):2104635. doi: 10.1002/advs.202104635 (PMC8948546; doi:10.1002/advs.202104635)
Supplement: Supplementary file 1 — Supporting Information [file ADVS-9-2104635-s001.pdf]

ADVANCED SCIENCE
Open Access

Supporting Information

for *Adv. Sci.*, DOI 10.1002/advs.202104635

Stretchable Sweat-Activated Battery in Skin-Integrated Electronics for Continuous Wireless Sweat Monitoring

Yiming Liu, Xingcan Huang, Jingkun Zhou, Chun Ki Yiu, Zhen Song, Wei Huang, Sina Khazaee Nejad, Hu Li, Tsz Hung Wong, Kuanming Yao, Ling Zhao, Woojung Yoo, Wooyoung Park, Jiyu Li, Ya Huang, Hiuwai Raymond Lam, Enming Song, Xu Guo, Yanwei Wang, Zhenxue Dai, Lingqian Chang*, Wen Jung Li*, Zhaoqian Xie* and Xinge Yu**

© 2020 Wiley-VCH GmbH

ADVANCED SCIENCE
Open Access

Supporting Information

for *Adv. Sci.,* DOI: 10.1002/advs.202104635

Stretchable Sweat-activated Battery in Skin-Integrated Electronics for Continuous Wireless Sweat Monitoring

Yiming Liu, Xingcan Huang, Jingkun Zhou, Chun Ki Yiu, Zhen Song, Wei Huang, Sina Khazaee Nejad, Hu Li, Tsz Hung Wong, Kuanming Yao, Ling Zhao, Woojung Yoo, Wooyoung Park, Jiyu Li, Ya Huang, Hiuwai Raymond Lam, Enming Song, Xu Guo, Yanwei Wang, Zhenxue Dai, Lingqian Chang*, Wen Jung Li*, Zhaoqian Xie*, and Xinge Yu**

© 2020 Wiley-VCH GmbH

Supplementary Information

Stretchable Sweat-activated Battery in Skin-Integrated Electronics for Continuous Wireless Sweat Monitoring

Yiming Liu[1†], Xingcan Huang[1†], Jingkun Zhou[1, 2†], Chun Ki Yiu[1, 2†], Zhen Song[3, 4†], Wei Huang[1], Sina Khazaee Nejad[1, 2], Hu Li[1], Tsz Hung Wong[1], Kuanming Yao[1], Ling Zhao[1], Woojung Yoo[1], Wooyoung Park[1], Jiyu Li[1, 2], Ya Huang[1, 2], Hiuwai Raymond Lam[1], Enming Song[2, 5], Xu Guo[3, 4], Yanwei Wang[6], Zhenxue Dai*[6], Lingqian Chang*[7], Wen Jung Li*[8], Zhaoqian Xie*[3, 4], and Xinge Yu*[1, 2]

[1]Department of Biomedical Engineering
City University of Hong Kong
Kowloon Tong 999077 (Hong Kong)
[2]Hong Kong Center for Cerebra-Cardiovascular Health Engineering
Hong Kong Science Park
New Territories 999077 (Hong Kong)
[3]State Key Laboratory of Structural Analysis for Industrial Equipment
Department of Engineering Mechanics
International Research Center for Computational Mechanics
Dalian University of Technology
Dalian 116024 (China)
[4]Ningbo Institute of Dalian University of Technology
Dalian University of Technology
Ningbo 315016 (China)
[5]Shanghai Frontiers Science Research Base of Intelligent Optoelectronics and Perception
Institute of Optoelectronics
Fudan University
Shanghai 200433 (China)
[6]College of Construction Engineering
Jilin University
Changchun 130012 (China)
[7]Beijing Advanced Innovation Center for Biomedical Engineering
School of Biological Science and Medical Engineering
Beihang University
Beijing 100191 (China)
[8]Department of Mechanical Engineering
City University of Hong Kong
Kowloon Tong (Hong Kong)

E-mail: dzx@jlu.edu.cn (ZD); lingqianchang@buaa.edu.cn (LC); wenjli@cityu.edu.hk (WL); zxie@dlut.edu.cn (ZX); xingeyu@cityu.edu.hk (XY)

Keywords: Sweat activated battery, sweat analysis, wireless communication, stretchable electronics, microfluidic system

Table S1. The electrical performances of recently reported stretchable power sources.

Powering type	Energy capacity	Power output	Demonstrations	Reference
SAB	42.5 mAh	7.46 mW/cm^2	Capable of continuously powering 120 LEDs, and self-developed wireless microelectronics for sweat analysis.	This work
Triboelectric nanogenerator	None	66.14 mW/m^2	Powering 250 LEDs	[1]
Triboelectric nanogenerator	None	95.4 mW/m^2	Powering a watch	[2]
Triboelectric nanogenerator	None	230 mW/m^2	Powering 170 LEDs	[3]
Piezoelectric nanogenerator	None	81.25 µW/cm^3	Powering a commercial calculator	[4]
Biofuel cells	None	3.5 mW/cm^2	Intermittently wireless sweat monitoring and human-machine interfaces	[5]
Biofuel cells	None	1.2 mW/cm^2	Light LED and a Bluetooth Low Energy radio	[6]
Photovoltaics	None	11.46 W/g	Cardiac signal recording	[7]
Photovoltaics	None	20 mA/cm^2 0.81 V	Self-powered strain sensors	[8]

Table S2. The reported fluid activated batteries over the past 10 years.

**Fluid**	**Flexibility**	**Functional Materials**	**Power density**	**Reference**
Blood	Flexible, and conformal	Aluminum, Silver Oxide	476.11 µW/cm^{2}µm	[9]
Simulated body fluids	Flexible, and folding	Znic, Polypyrrode-carbon nanotube composites	60 µA/cm^2	[10]
Sweat	Flexible	Mg, Ag/AgCl	~580 Wh/kg	[11]
Buffer	Flexible	Mg, AgNO3	3 mW/cm^2	[12]
Sweat	Flexible, and	Mg, Ag/AgCl	2 mW	[13]

	conformal			
Body Fluid	Rigid	Mg, Fe	0.1 mA/cm^2	[14]
**Sweat**	**Stretchable**	**Zn, $CuSO_4$**	**7.46 mW/cm^2**	**This work**

Table S3. Average estimated SAB activated time mounted onto human subjects as the users are doing various exercises[15].

User with different professions	Age	Body weight (kg)	Exercise duration (h)	Whole body sweating rate (L/h)	**Estimated battery activated time (min)**
Individual power athletes	24 ± 3	108.1 ± 23.9	1.1 ± 0.3	0.88 ± 0.25	**48.82**
Fitness athletes	30 ± 4	66.1 ± 14.9	1.3 ± 0.2	1.05 ± 0.50	**40.91**
Action athletes	25 ± 5	70.4 ± 10.5	1.5 ± 0.9	0.90 ± 0.50	**47.73**
Endurance	35 ± 1	70.3 ± 9.8	1.2 ± 0.7	1.28 ± 0.57	**33.56**
Team/skill sport athletes	21 ± 5	87.8 ± 24.6	1.9 ± 0.7	1.10 ± 0.58	**39.05**
Baseball	22 ± 4	88.6 ± 12.4	2.0 ± 0.8	0.83 ± 0.34	**51.76**
Basketball	23 ± 5	92.1 ± 18.0	2.1 ± 0.8	0.95 ± 0.42	**45.22**
American football	24 ± 4	111.6 ± 23.2	2.1 ± 0.6	1.51 ± 0.70	**28.45**
Soccer	18 ± 6	65.7 ± 15.4	1.5 ± 0.3	0.94 ± 0.38	**45.70**

Table S4. Sweat rate in men and women during moderate exercise-heat stress[16], and corresponding estimate SAB activated time.

Area	Men Sweating Rate, mL.cm^{-2}.min^{-1}	**Male Estimated battery activated time (min)**	Women Sweating Rate, mL.cm^{-2}.min^{-1}	**Female Estimated battery activated time (min)**
Whole body	$(0.712 \pm 0.173)\times10^{-3}$	56.18	$(0.610 \pm 0.139)\times10^{-3}$	65.57
Dorsal forearm	$(1.562 \pm 0.603)\times10^{-3}$	25.61	$(1.313 \pm 0.497)\times10^{-3}$	30.46
Ventral forearm	$(1.603 \pm 0.510)\times10^{-3}$	24.95	$(1.240 \pm 0.357)\times10^{-3}$	32.26
Triceps	$(1.273 \pm 0.623)\times10^{-3}$	31.42	$(0.916 \pm 0.306)\times10^{-3}$	43.67
Chest	$(1.555 \pm 0.636)\times10^{-3}$	25.72	$(1.127 \pm 0.425)\times10^{-3}$	35.49
Scapula	$(2.036 \pm 0.771)\times10^{-3}$	19.65	$(1.509 \pm 0.590)\times10^{-3}$	26.57
Lower back	$(1.800 \pm 0.556)\times10^{-3}$	22.22	$(1.556 \pm 0.775)\times10^{-3}$	25.71
Ventral thigh	$(1.030 \pm 0.282)\times10^{-3}$	38.83	$(0.866 \pm 0.374)\times10^{-3}$	46.19
Calf	$(0.765 \pm 0.344)\times10^{-3}$	52.29	$(0.731 \pm 0.349)\times10^{-3}$	54.72
Forehead	$(5.931 \pm 3.005)\times10^{-3}$	6.74	$(2.433 \pm 1.319)\times10^{-3}$	16.44
9-Site	$(1.644 \pm 0.515)\times10^{-3}$	24.33	$(1.171 \pm 0.405)\times10^{-3}$	34.16

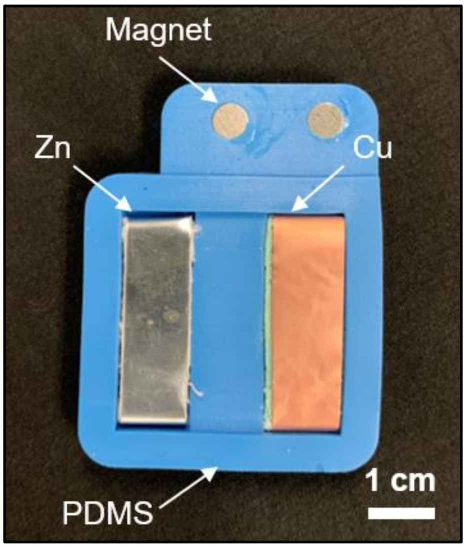

Fig. S1. Optical image of the inner view of the sweat activated battery cell.

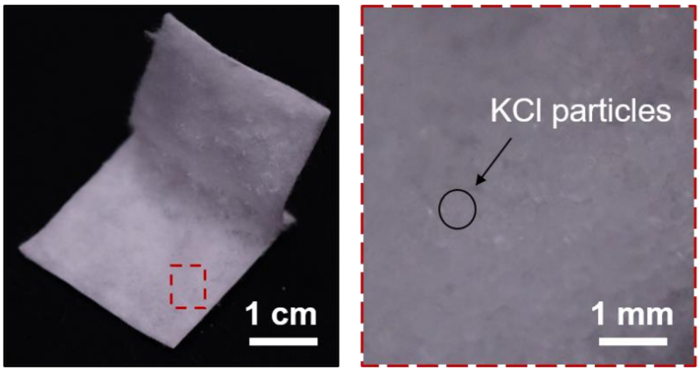

Fig. S2. Optical images of the water absorption cotton with KCl powder insides.

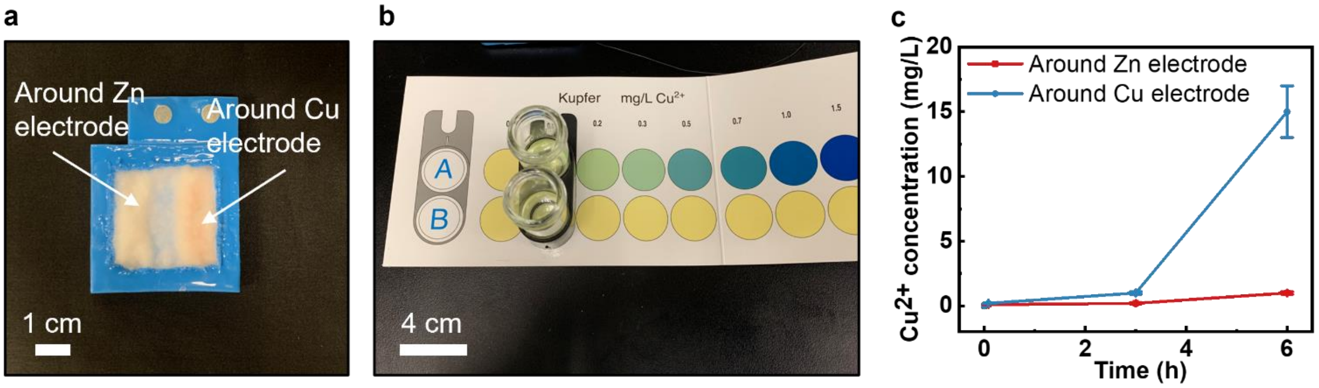

Fig. S3. The Cu^{2+} concentration test around the SAB with 0.7 mL/cm^2 artificial sweat injected. (a) Optical image of the SAB as 0.7 mL/cm^2 artificial sweat has been injected for 6 hrs. (b) The experiment setup of the Cu^{2+} concentration test. (c) The Cu^{2+} concentrations around Zn and Cu electrode as a function of the battery operation time. Here the battery has been powering a constant resistance, 180 Ω.

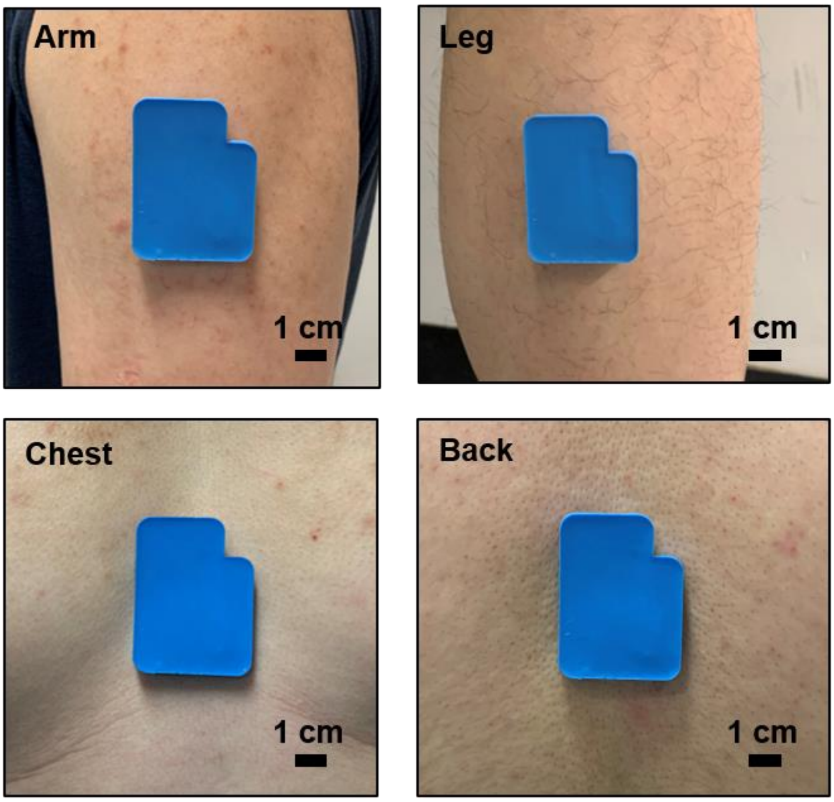

Fig. S4. Optical images of the sweat activated battery cell mounted onto human body, including arm, back, and chest.

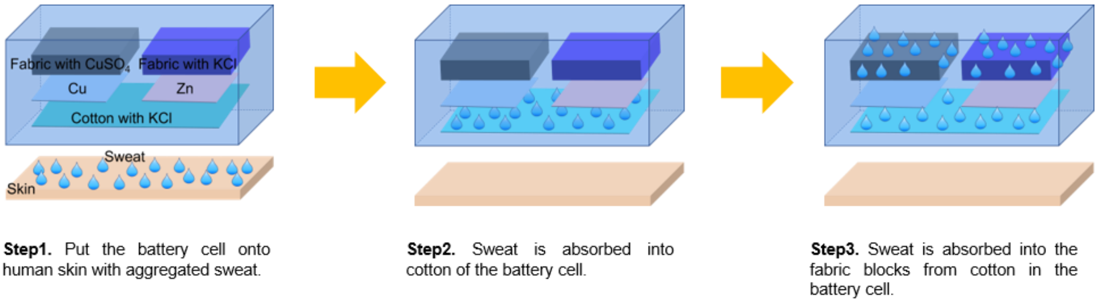

Fig. S5. Schematic diagram of the working principle of the sweat activated battery cell.

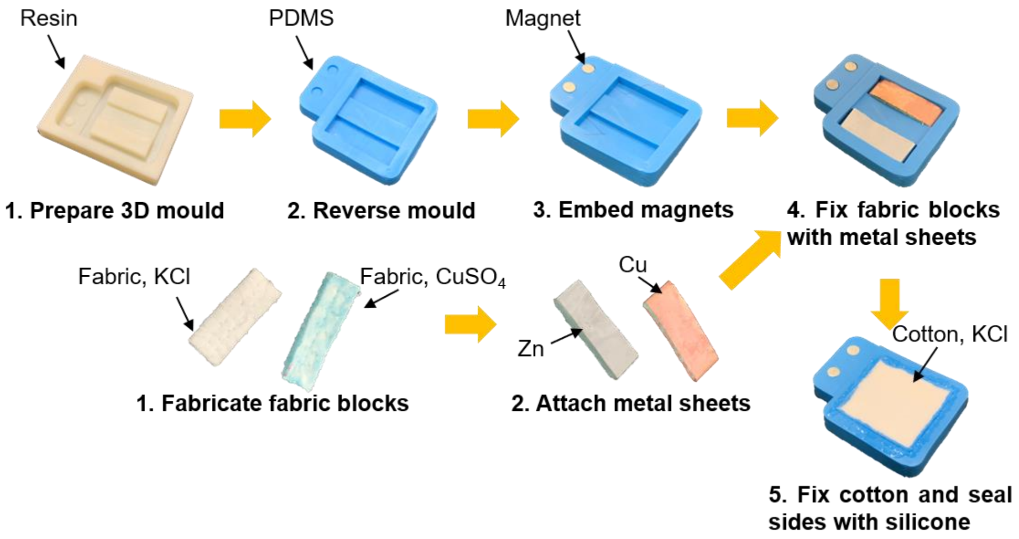

Fig. S6. Schematic diagram of the fabrication process of the sweat activated battery cell.

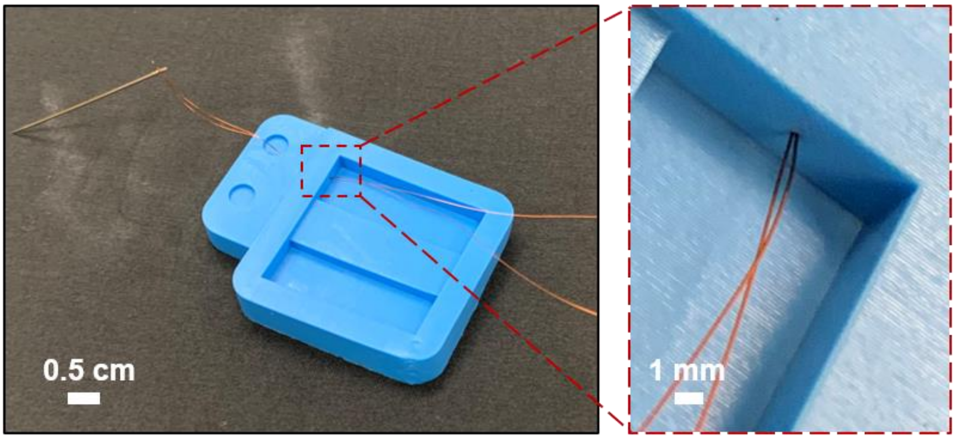

Fig. S7. Optical images of the battery cell encapsulation, showing that a needle is used to embed wires for later connecting magnets and working metal sheets (Cu, and Zn).

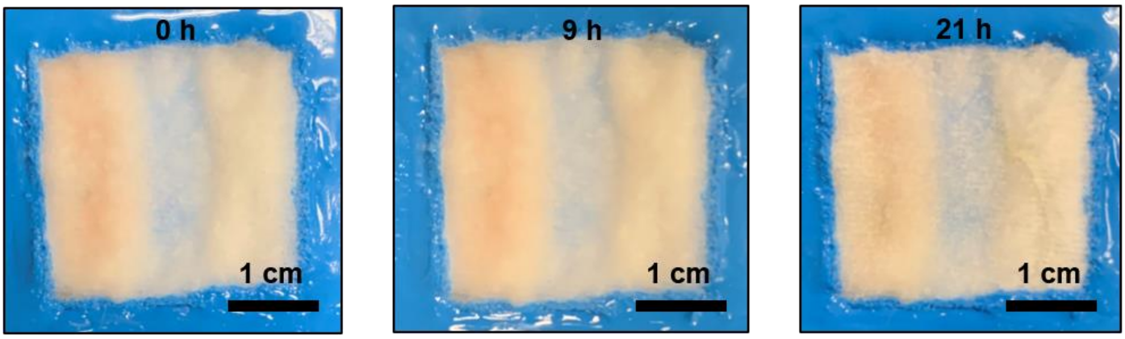

Fig. S8. Optical images of the sweat activated battery cell injected 0.6 mL artificial sweat for over 21 h. Here, it is obvious that the cotton can perfectly prevent the chemicals leakage from the inner space of the battery as the battery is injected 1 mL artificial sweat.

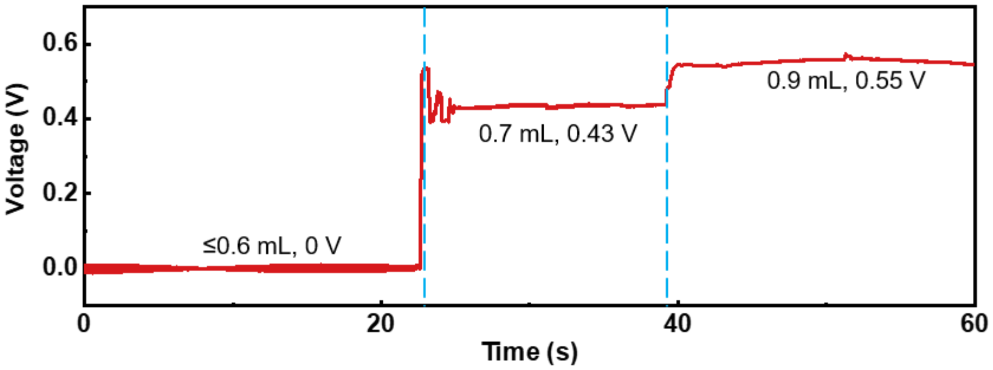

Fig. S9. Electrical response of one battery cell without any ionic chemicals in cotton as a function of time as added artificial sweat gradually from 0 to 0.9 mL.

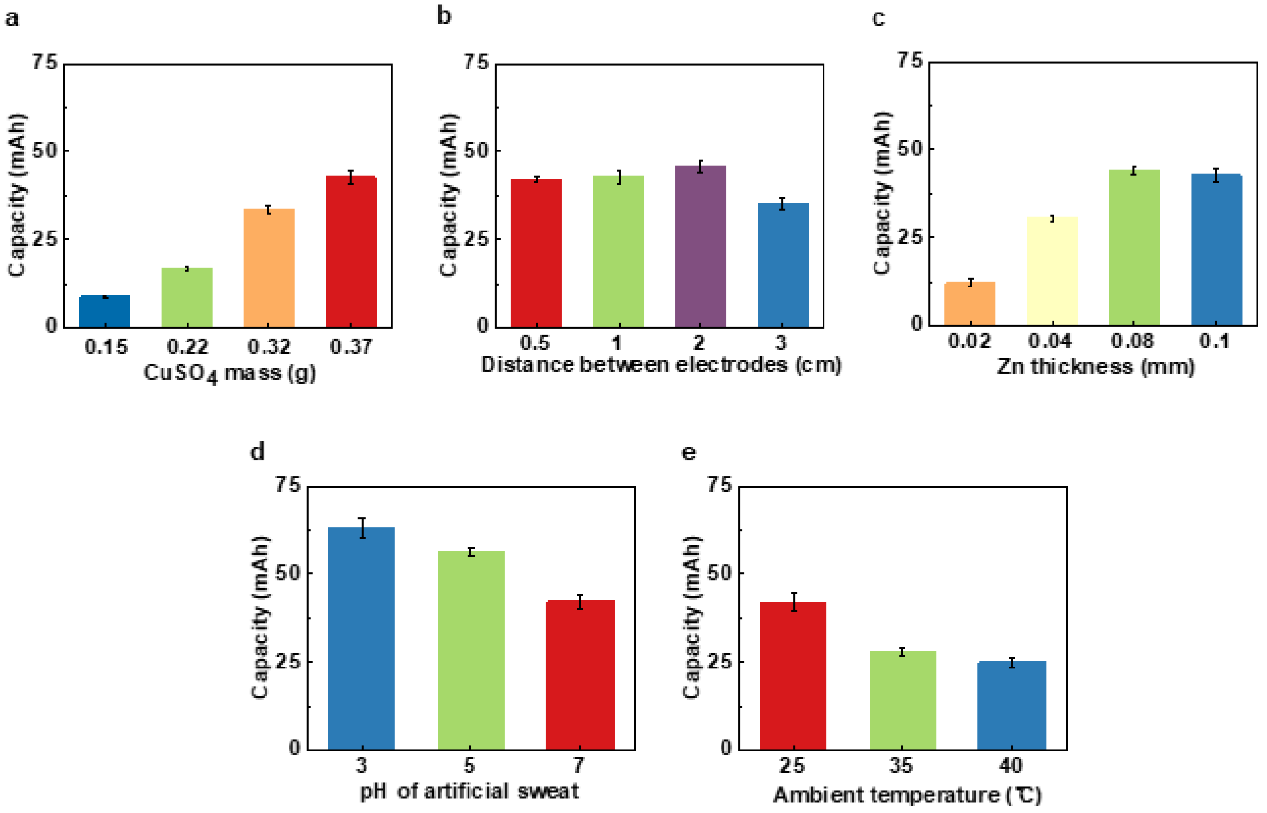

Fig. S10. Capacity of the sweat activated battery as a function of $CuSO_4$ mass in the fabric block (**a**), distance between the two electrodes (**b**), thickness of Zn sheet (**c**), pH of the artificial sweat (**d**), and ambient temperature under the neutral condition (pH = 7) (**e**).

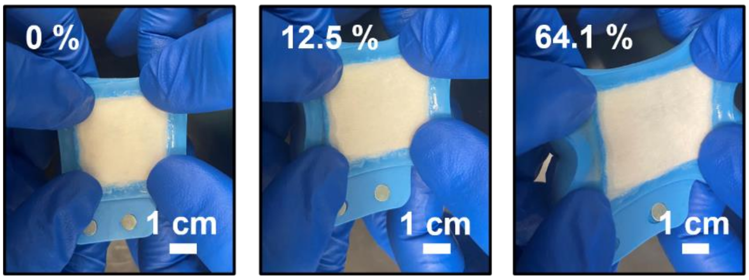

Fig. S11. Optical images of the sweat activated battery under various stretching conditions, including 0, 12.5%, and 64.1%.

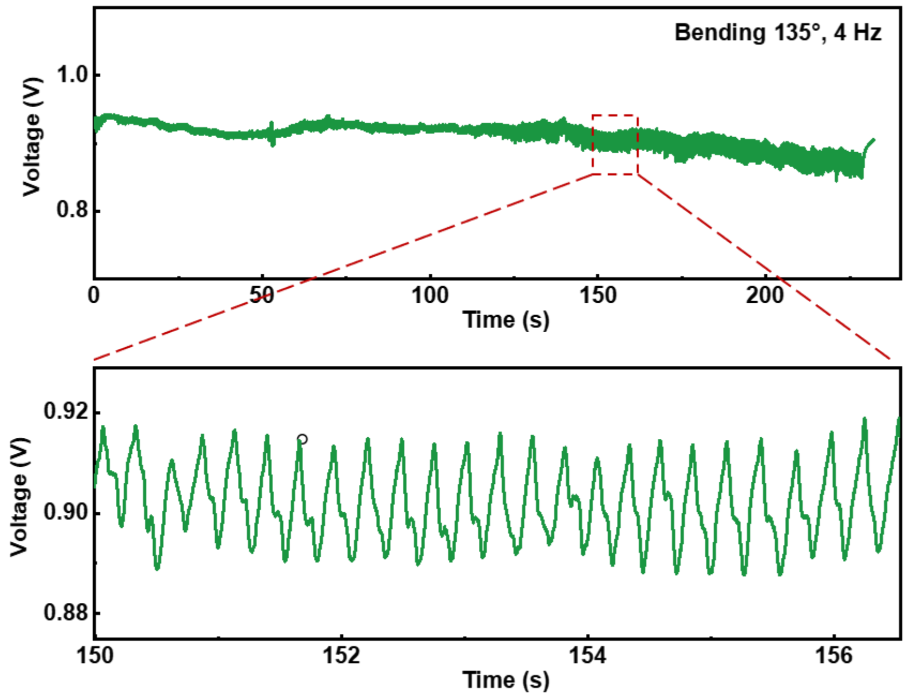

Fig. S12. Electrical response of the sweat activated battery under a bending angle and frequency of 135° and 4 Hz, respectively.

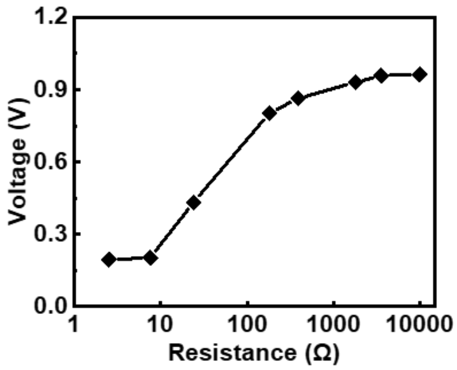

Fig. S13. Voltage output of one battery cell as a function of connected resistance from 2.5 Ω to 10 kΩ.

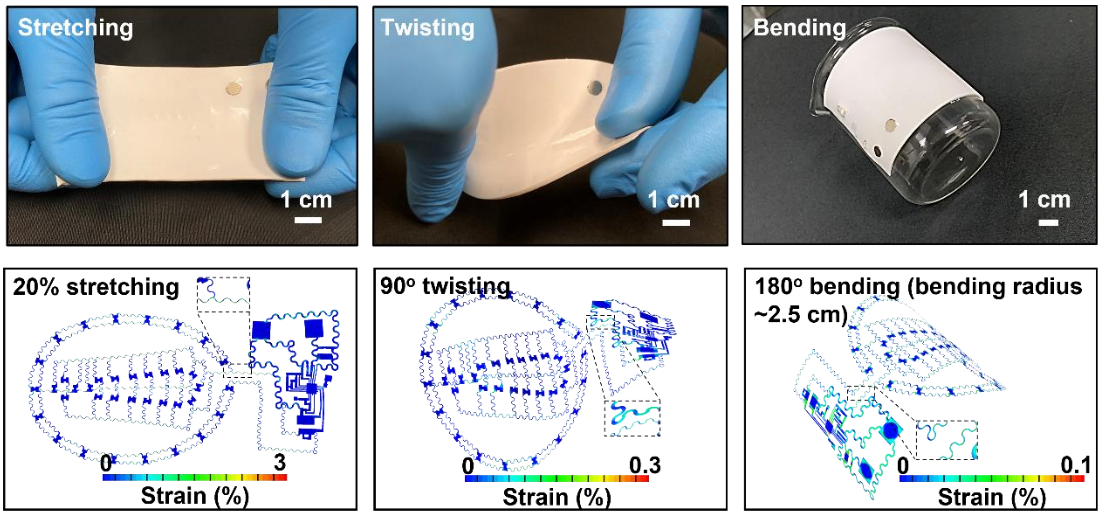

Fig. S14. Optical images and FEA models of the lighting electronics under the three different mechanical deformations, including stretching, twisting, and bending.

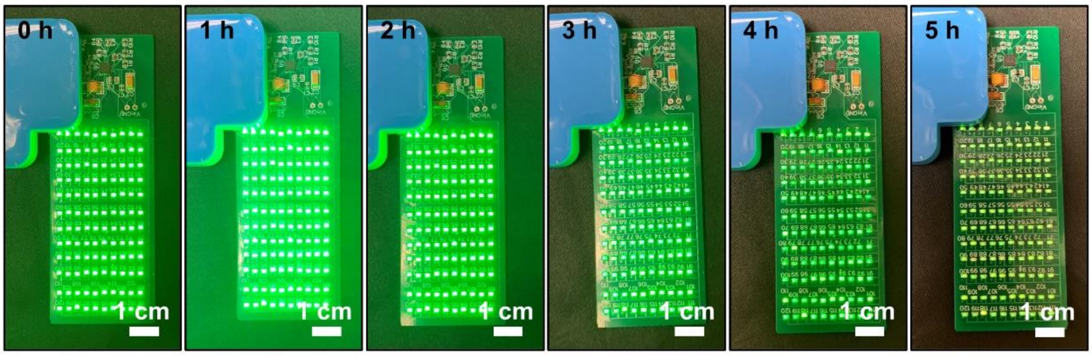

Fig. S15. Optical images of the 120-LED electronics powered by the flexible battery at 0 h, 1 h, 2 h, 3 h, 4 h, and 5 h.

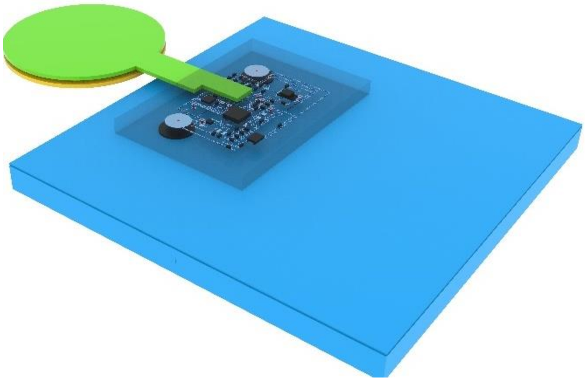

Fig. S16. Schematic diagram of the sweat electronics powered by the four integrated sweat activated battery cells.

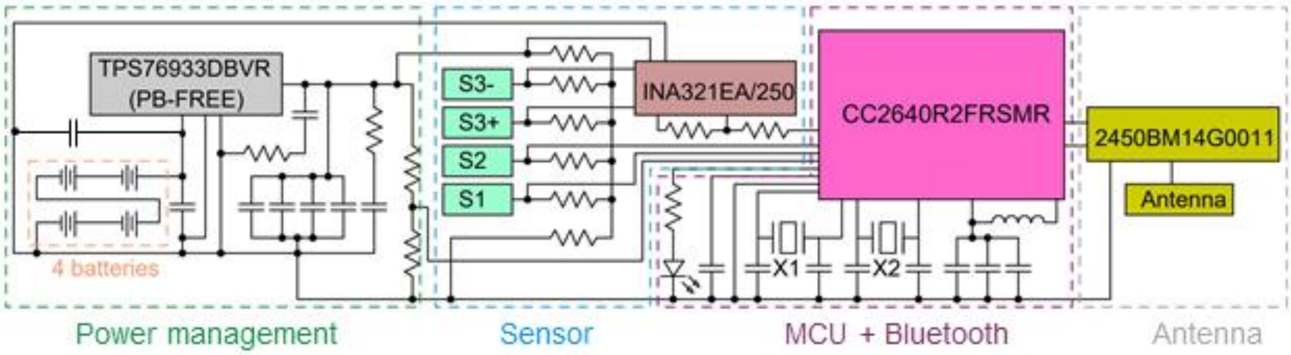

Fig. S17. The circuit design of the sweat microelectronics.

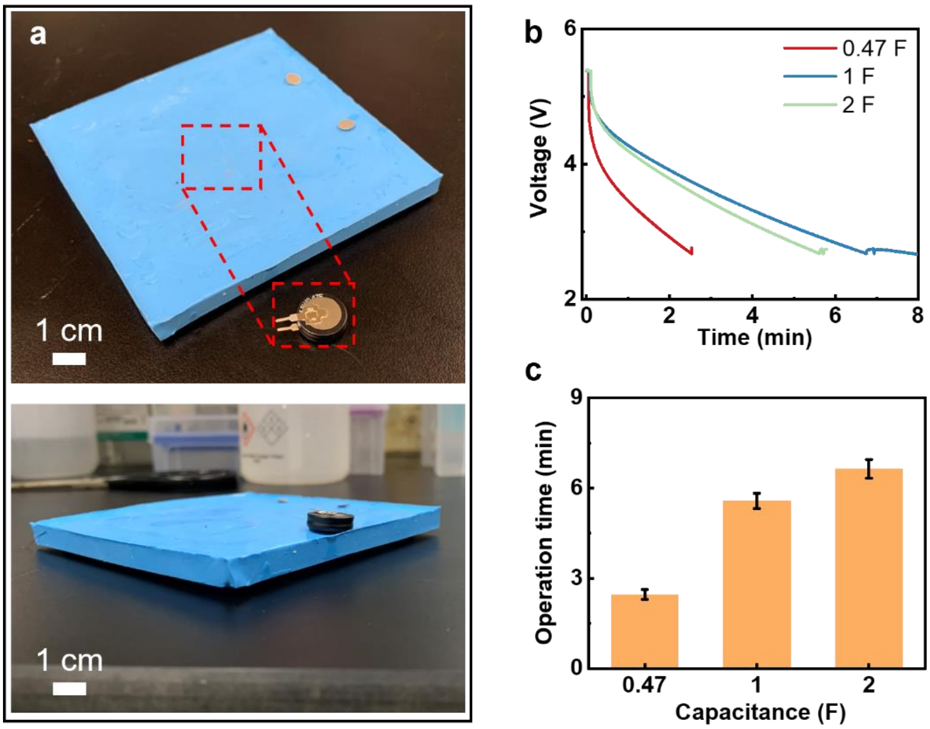

Fig. S18. Discharging curves of three supercapacitors in powering the microelectronics. (a) Optical images of the SABs with supercapacitors embedded. (b) Electrical response of three supercapacitors powering the microelectronics. (c) Operation time of the microelectronics powered by the three supercapacitors.

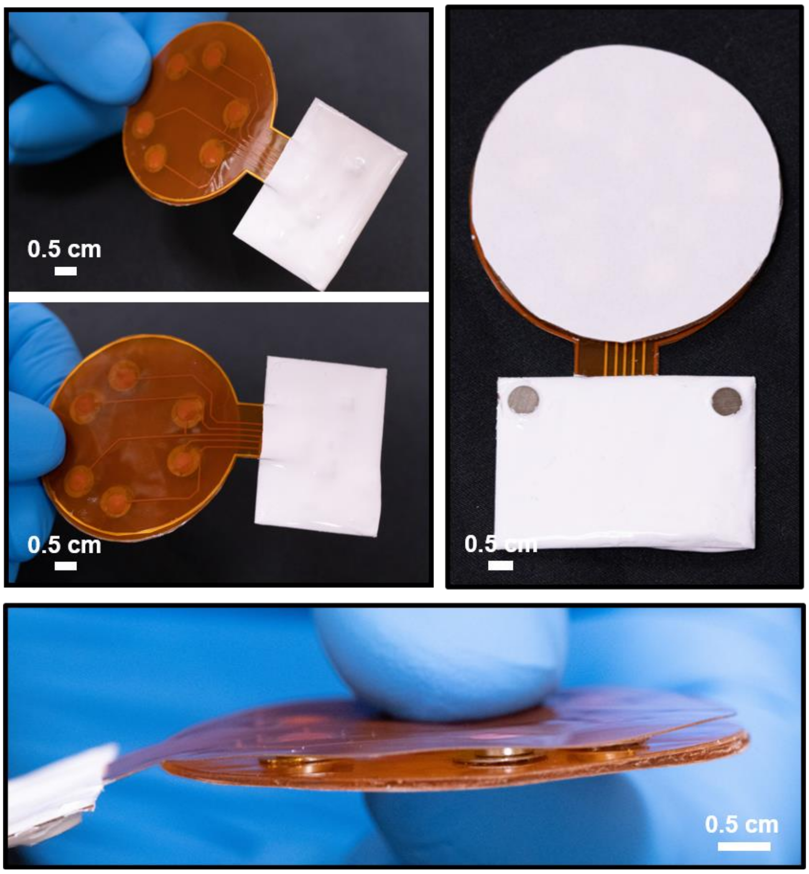

Fig. S19. Optical images of the sweat microelectronics.

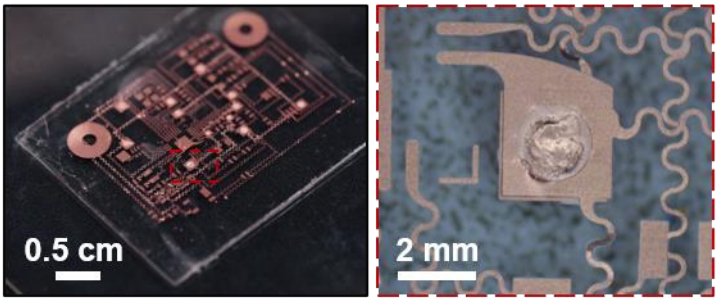

Fig. S20. Optical images of the Cu based circuit of the sweat microelectronics with the enlarged detain.

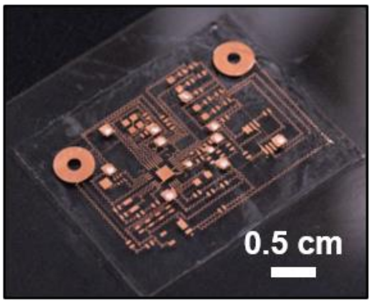

Fig. S21. Optical image of the Cu based circuit with vertical bridge of the sweat microelectronics.

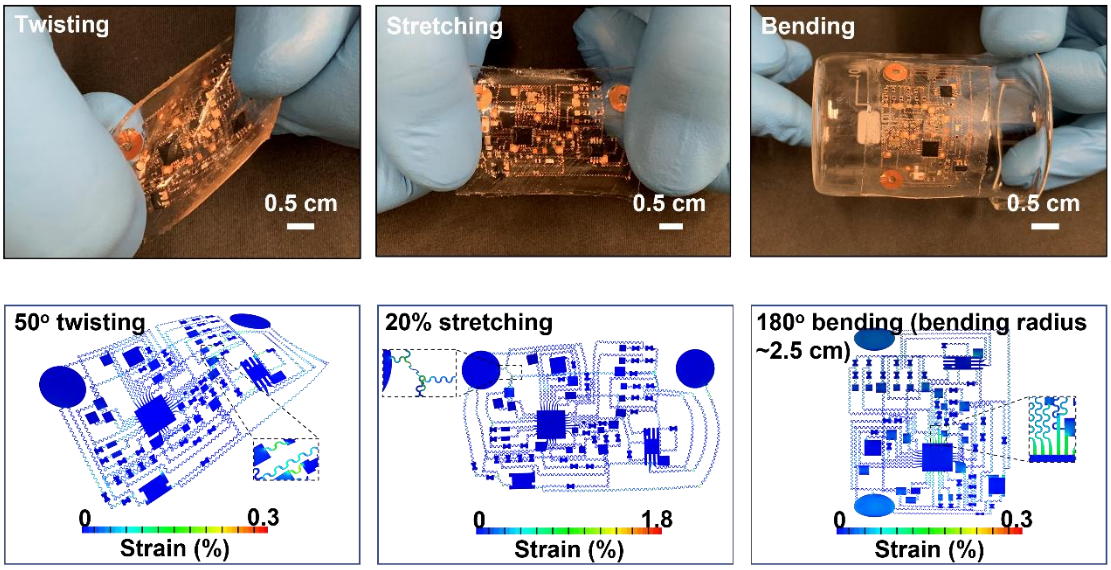

Fig. S22. Optical images and FEA models of the sweat microelectronics under the three different mechanical deformations, including twisting, stretching, and bending.

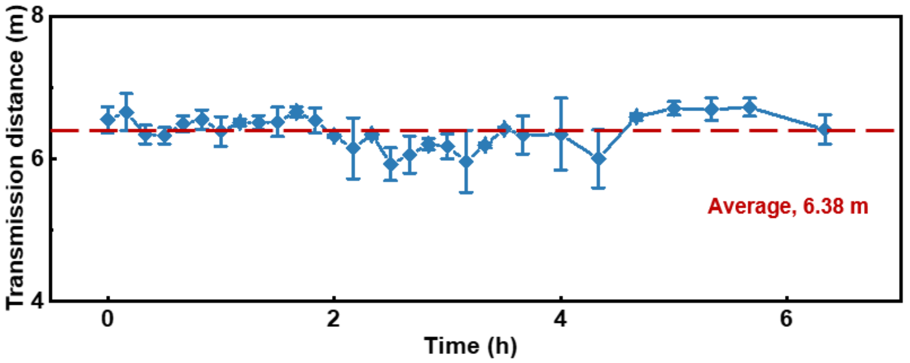

Fig. S23. Transmission distance of the sweat electronics, powered by the four integrated sweat activated battery cells, as a function of time as mounted onto human back for over 6 h.

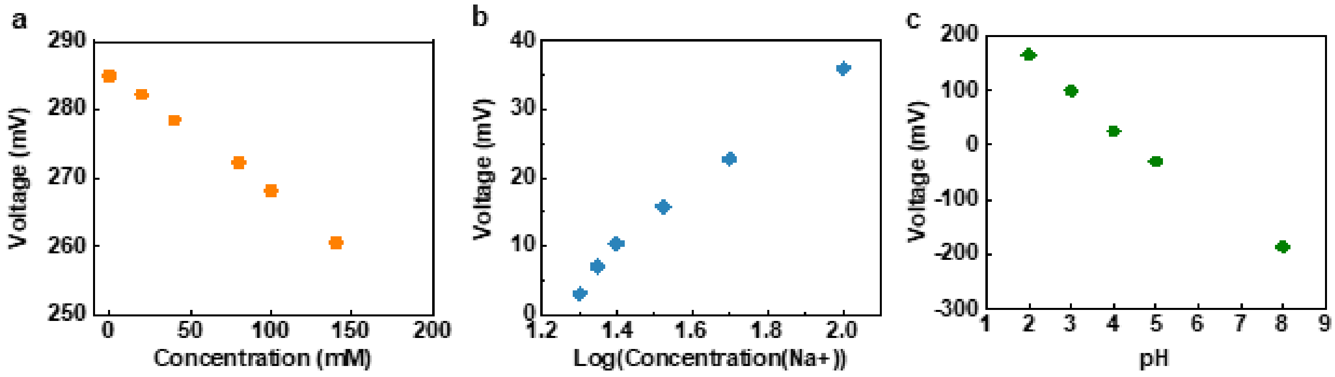

Fig. S24. Open circuit voltage responses of glucose, Na^+ and pH sensors.

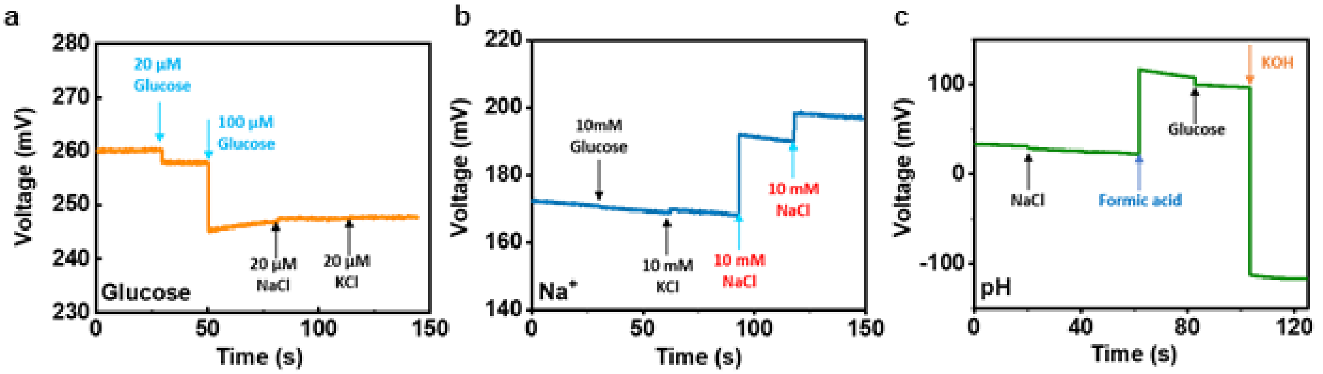

Fig. S25. Anti-interference capabilities of glucose, Na^+, and pH biosensors.

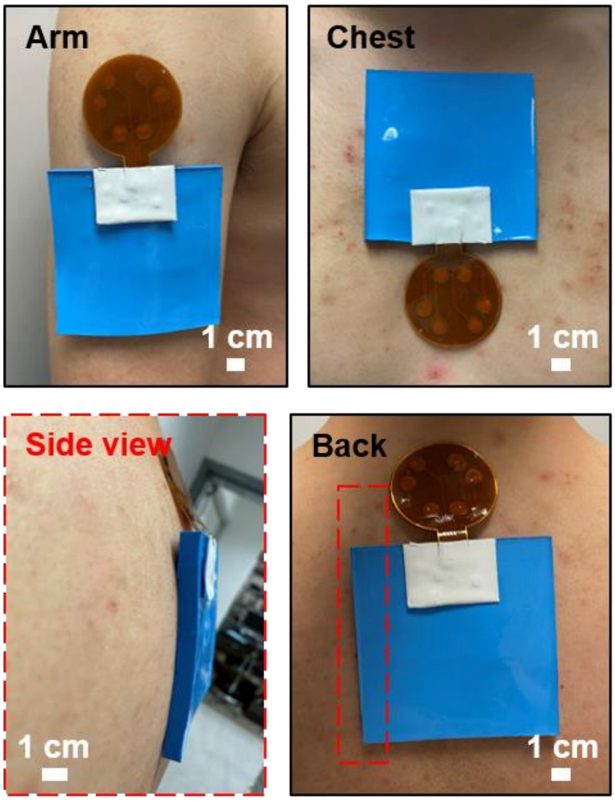

Fig. S26. Optical images of the sweat microelectronics with the four integrated battery cells mounted onto human body, including arm, chest, and back.

Reference

1 Xu, F. *et al.* Scalable fabrication of stretchable and washable textile triboelectric nanogenerators as constant power sources for wearable electronics. *Nano Energy*, 106247 (2021).

2 Cong, Z. *et al.* Stretchable coplanar self-charging power textile with resist-dyeing triboelectric nanogenerators and microsupercapacitors. *ACS nano* **14**, 5590-5599 (2020).

3 Dong, K. *et al.* A stretchable yarn embedded triboelectric nanogenerator as electronic skin for biomechanical energy harvesting and multifunctional pressure sensing. *Advanced Materials* **30**, 1804944 (2018).

4 Niu, X. *et al.* High-performance PZT-based stretchable piezoelectric nanogenerator. *ACS Sustainable Chemistry & Engineering* **7**, 979-985 (2018).

5 Yu, Y. *et al.* Biofuel-powered soft electronic skin with multiplexed and wireless sensing for human-machine interfaces. *Sci Robot* **5**, doi:10.1126/scirobotics.aaz7946 (2020).

6 Bandodkar, A. J. *et al.* Soft, stretchable, high power density electronic skin-based biofuel cells for scavenging energy from human sweat. *Energ Environ Sci* **10**, 1581-1589, doi:10.1039/c7ee00865a (2017).

7 Park, S. *et al.* Self-powered ultra-flexible electronics via nano-grating-patterned organic photovoltaics. *Nature* **561**, 516-521, doi:10.1038/s41586-018-0536-x (2018).

8 Dauzon, E. *et al.* Stretchable and Transparent Conductive PEDOT:PSS-Based Electrodes for Organic Photovoltaics and Strain Sensors Applications. *Adv Funct Mater* **30**, doi:10.1002/adfm.202001251 (2020).

9 Garay, E. F. & Bashirullah, R. Biofluid activated microbattery for disposable microsystems. *Journal of Microelectromechanical Systems* **24**, 70-79 (2014).

10 Li, S., Guo, Z. P., Wang, C. Y., Wallace, G. G. & Liu, H. K. Flexible cellulose based polypyrrole–multiwalled carbon nanotube films for bio-compatible zinc batteries activated by simulated body fluids. *Journal of Materials Chemistry A* **1**, 14300-14305 (2013).

11 Bandodkar, A. *et al.* Sweat-activated biocompatible batteries for epidermal electronic and microfluidic systems. *Nature Electronics* **3**, 554-562 (2020).

12 Koo, Y., Sankar, J. & Yun, Y. High performance magnesium anode in paper-based microfluidic battery, powering on-chip fluorescence assay. *Biomicrofluidics* **8**, 054104 (2014).

13 Ortega, L., Llorella, A., Esquivel, J. P. & Sabaté, N. Self-powered smart patch for sweat conductivity monitoring. *Microsystems & nanoengineering* **5**, 1-10 (2019).

14 She, D., Tsang, M. & Allen, M. Biodegradable batteries with immobilized electrolyte for transient MEMS. *Biomedical microdevices* **21**, 1-9 (2019).

15 Barnes, K. A. *et al.* Normative data for sweating rate, sweat sodium concentration, and sweat sodium loss in athletes: An update and analysis by sport. *Journal of Sports Sciences* **37**, 2356-2366 (2019).

16 Baker, L. B. *et al.* Body map of regional vs. whole body sweating rate and sweat electrolyte concentrations in men and women during moderate exercise-heat stress. *Journal of Applied Physiology* **124**, 1304-1318, doi:10.1152/japplphysiol.00867.2017 (2018).
